# Selenoprotein K deficiency inhibits melanoma by reducing calcium flux required for tumor growth and metastasis

**DOI:** 10.18632/oncotarget.24388

**Published:** 2018-02-03

**Authors:** Michael P. Marciel, Vedbar S. Khadka, Youpeng Deng, Pascal Kilicaslan, Andrew Pham, Pietro Bertino, Katie Lee, Suzie Chen, Natalija Glibetic, FuKun W. Hoffmann, Michelle L. Matter, Peter R. Hoffmann

**Affiliations:** ^1^ Department of Cell and Molecular Biology, John A. Burns School of Medicine, University of Hawaii, Honolulu, Hawaii, U.S.A.; ^2^ Bioinformatics Core in the Department of Complementary and Integrative Medicine, John A. Burns School of Medicine, University of Hawaii, Honolulu, Hawaii, U.S.A.; ^3^ Biotechnology Department, University of Applied Sciences Mannheim, Mannheim, Germany; ^4^ Ernest Mario School of Pharmacy, Rutgers University, Piscataway, New Jersey, U.S.A.; ^5^ The University of Hawaii Cancer Center, Honolulu, Hawaii, U.S.A.

**Keywords:** selenium, migration, inositol 1,4,5-triphosphate receptor, calcium channel, palmitoylation

## Abstract

Interest has emerged in the therapeutic potential of inhibiting store operated calcium (Ca^2+^) entry (SOCE) for melanoma and other cancers because malignant cells exhibit a strong dependence on Ca^2+^ flux for disease progression. We investigated the effects of deleting Selenoprotein K (SELENOK) in melanoma since previous work in immune cells showed SELENOK was required for efficient Ca^2+^ flux through the endoplasmic reticulum Ca^2+^ channel protein, inositol 1,4,5-trisphosphate receptor (IP3R), which is due to the role SELENOK plays in palmitoylating and stabilizing the expression of IP3R. CRISPR/Cas9 was used to generate SELENOK-deficiency in human melanoma cells and this led to reduced Ca^2+^ flux and impaired IP3R function, which inhibited cell proliferation, invasion, and migration. Ca^2+^-dependent signaling through calcineurin was inhibited with SELENOK-deficiency, and gene array analyses together with evaluation of transcript and protein levels showed altered transcriptional programs that ultimately disrupted stemness and pro-growth properties. *In vivo* investigations were conducted using the Grm1-Tg transgenic mouse strain that develops spontaneous metastatic melanoma, which was crossed with SELENOK^−/−^ mice to generate the following littermates: Grm1-Tg/SELENOK^−/−^, Grm1-Tg/SELENOK^−/+^, Grm1-Tg/SELENOK^+/+^. SELENOK-deficiency in Grm1-Tg/SELENOK^−/−^ male and female mice inhibited primary tumor growth on tails and ears and reduced metastasis to draining lymph nodes down to levels equivalent to non-tumor control mice. Cancer stem cell pools were also decreased in Grm1-Tg/SELENOK^−/−^ mice compared to littermates. These results suggest that melanoma requires SELENOK expression for IP3R dependent maintenance of stemness, tumor growth and metastasic potential, thus revealing a new potential therapeutic target for treating melanoma and possibly other cancers.

## INTRODUCTION

Calcium (Ca^2+^) is a ubiquitous signaling ion and intracellular second messenger that is critical to a variety of cellular processes. The primary store of intracellular Ca^2+^ is the endoplasmic reticulum (ER), and cytosolic Ca^2+^ is maintained at nanomolar levels by plasma membrane Ca^2+^ ATPases and SR/ER Ca^2+^ ATPases that pump Ca^2+^ into the extracellular space and the ER lumen, respectively [1]. Agonist activation of a variety of surface receptors activates phospholipase C that subsequently catalyzes the hydrolysis of phosphatidylinositol 4,5-bisphosphate to diacyl glycerol and inositol 1,4,5-trisphosphate (IP3). IP3 acts upon ER-membrane IP3 receptors (IP3Rs) to release Ca^2+^ from the ER lumen, which is sensed by the EF-hand motif of stromal interaction molecules (STIM1 and STIM2) within the ER lumen [2]. This induces structural changes in STIM that promotes its oligomerization and translocation to the ER membrane domains proximal to the plasma membrane, where it triggers the opening of the Orai Ca^2+^ channel in the plasma membrane [3]. This process is termed store operated Ca^2+^ entry (SOCE) [4], and is operational in a wide variety of different cell types for activating Ca^2+^ dependent signaling cascades that in turn are required for cellular functions such as proliferation and migration [5].

When different cell-types transform into malignant tumor cells, Ca^2+^ homeostasis and signaling may be altered in a process referred to as Ca^2+^ remodeling [6]. SOCE and the downstream Ca^2+^ dependent signaling cascades are exploited by cancer cells to increase their capacity to proliferate, invade and migrate. Because SOCE is important for both primary tumor progression and metastasis to other tissues, molecules regulating SOCE may serve as important targets for developing anti-cancer therapeutics [7, 8]. In fact, cancer cells may develop a stringent dependence on highly efficient SOCE along with the signaling pathways and gene expression programs shaped by Ca^2+^ remodeling. However, there is limited information regarding targetable mechanisms that drive SOCE in malignant melanoma, supporting the need for better insight and potential therapeutic targets in the SOCE pathway.

Our previous work that focused on immune cells led to identification of a mechanism regulating Ca^2+^ flux through the actions of an ER transmembrane protein, selenoprotein K (SELENOK), which is a member of the selenoprotein family that contains 25 members that each contain the 21st amino acid, selenocysteine. Expression levels of SELENOK are regulated by dietary selenium [9, 10] and its function is regulated in some cell types by calpain cleavage [11]. The generation of SELENOK knockout mice revealed a role for SELENOK in promoting Ca^2+^ flux during receptor driven activation of immune cells [9]. We subsequently uncovered the mechanism by which SELENOK regulates Ca^2+^ flux by demonstrating its role as a cofactor that binds to the palmitoyl acyl transferase, DHHC6, to catalyze the palmitoylation of the IP3R [12]. Without the SELENOK/DHHC6 catalyzed addition of this lipid moiety, the tetrameric IP3R Ca^2+^ channel in the ER membrane is unstable and degraded through the proteasome and Ca^2+^ flux is impaired [13]. Herein, we provide evidence that SELENOK is indeed required for effective Ca^2+^ flux in human melanoma cells and SELENOK deficiency changes their growth and stemness properties, leading to poor proliferation and migration *in vitro* and *in vivo*.

## RESULTS

### SELENOK is important for melanoma cell proliferation

**Figure 1:**
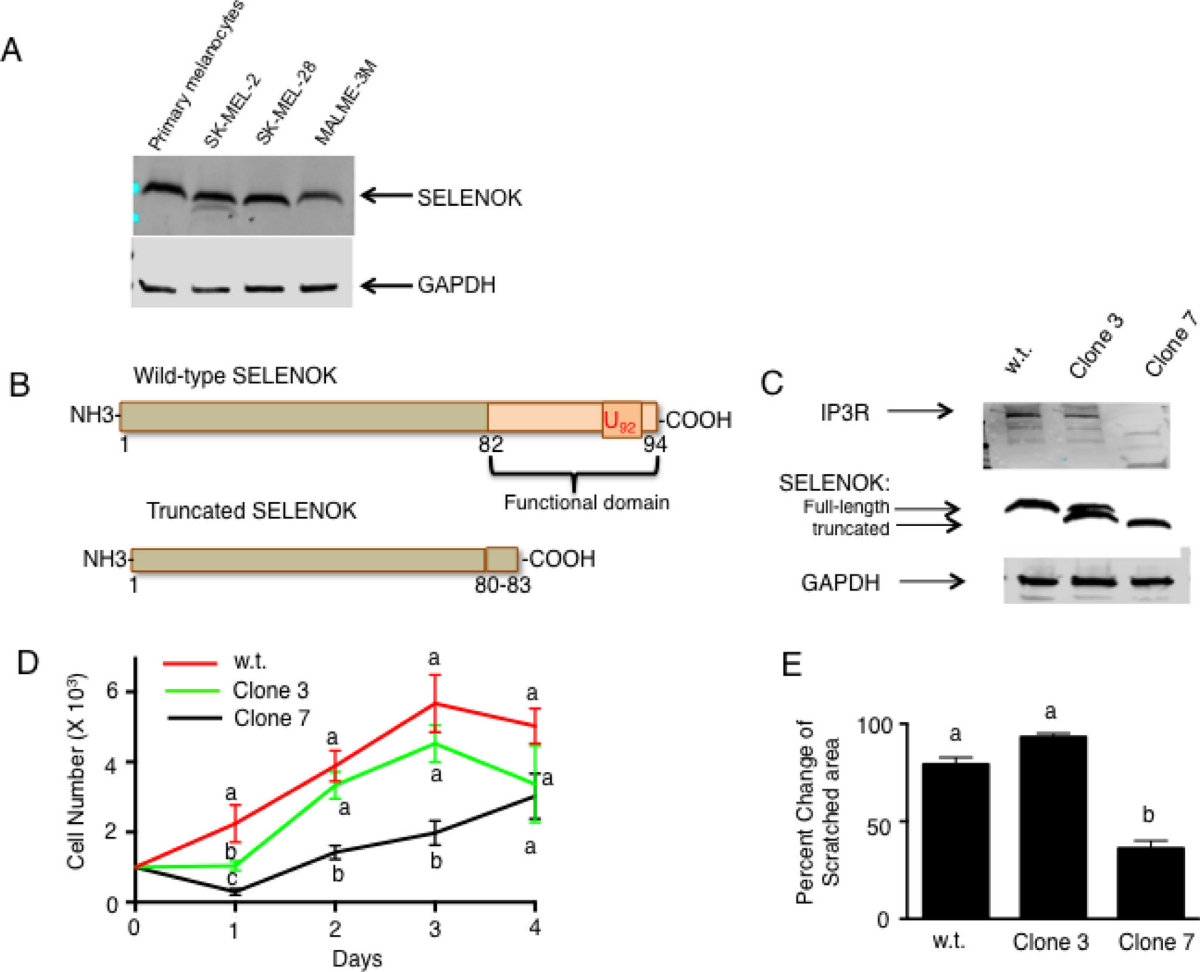
Loss of functional SELENOK in melanoma cells leads to decreased proliferation (**A**) Western blot analysis showed similar SELENOK levels in primary human melanocytes and three human melanoma cell lines. GAPDH was used as a loading control. (**B**) A diagram illustrates how CRISPR/Cas9 was used to edit the genome of SK-MEL-28 cells, generating a truncated version of SELENOK with its functional domain deleted. (**C**) Western blot confirmed presence of full-length SELENOK in w.t. cells, both full-length and truncated SELENOK in Clone 3, but only truncated SELENOK in Clone 7 cells. Only Clone 7 exhibited reduced IP3R levels. GAPDH was used as a loading control. (**D**) Equal numbers of cells were plated in replicate wells (*N* = 5 per cell line) and proliferation was measured over a 4-day period. Clone 7 showed reduced growth on days 1–3. Results are expressed as mean + SEM and a one-way ANOVA with Tukey post-test was used to analyze groups. Means at each time point without a common letter differ, *p* < 0.05. (**E**) Scratch assays were performed in triplicate comparing w.t. SK-MEL-28 cells to Clone 3 and 7 cells. Results showed less enclosure of the scratched region for Clone 7 cells. For D–E, results represent two independent experiments and a one-way ANOVA was used to analyze groups with Tukey post-test used to compare means of each group. Results are expressed as mean + SEM and means without a common letter differ, *p* < 0.05.

In some cases, but not all, molecules involved in SOCE are found at higher levels in melanoma tumor tissues compared to healthy tissues [14]. We examined SELENOK levels in early and late stage malignant melanoma tissues from a small group of patients (*N* = 10) and found no differences compared to normal control tissues (Supplementary Figure 1). We also evaluated SELENOK levels in three NCI-60 validated human melanoma cell lines (SK-MEL-2, SK-MEL-28, MALME-3M) along with primary melanocyte lysate as a control. Consistent with the tissue data described above, equivalent levels of SELENOK were found in primary melanocytes compared to the melanoma cell lines (Figure 1A). These data suggest that SELENOK is expressed in melanoma cells but its levels may not be increased compared to normal tissues. Our data also suggested that these human cell lines may be useful for SELENOK loss-of-function studies and this was our next course of action.

Because SELENOK is required for the post-translational palmitoylation of the IP3R and stable configuration of this Ca^2+^ channel in the ER membrane that allows efficient SOCE in immune cells [13], we hypothesized that SELENOK-deficient melanoma cells would exhibit impaired growth that depends on efficient Ca^2+^ flux. As shown in Figure 1B, CRISPR/Cas9 was used to edit the SELENOK gene in SK-MEL-28 cells to generate clones expressing truncated SELENOK lacking the C-terminal functional domain of SELENOK [11]. Two clones generated from this approach were identified and expanded to produce stable cell lines as determined by western blot analyses (Figure 1C). One cell line contained an edited allele encoding truncated SELENOK and one unedited allele encoding full-length SELENOK (Clone 3), and another cell line had both alleles edited to generate only truncated SELENOK (Clone 7). These western blot results were consistent with Sanger sequencing of the clones (Supplementary Figure 2). Importantly, low levels of the Ca^2+^ channel protein, IP3R, were found in SELENOK-deficient Clone 7 cells, which is consistent with previous findings showing that SELENOK deficiency leads to reduced levels of IP3R in immune cells [12]. Proliferation assays were used to compare wild-type (w.t.) SK-MEL-28 to Clones 3 and 7 cells. Clone 7 cells proliferated at a lower rate compared to w.t. and Clone 3 cells, which were equivalent to each other (Figure 1D). Scratch assays were performed to determine ability of each cell line to grow and move into the scratched area, and results showed Clone 7 cells filled the scratched area at lower levels compared to w.t. and Clone 3 cells, which were equivalent to each other (Figure 1E).

### SELENOK deficient melanoma cells exhibit lower Ca^2+^ flux and altered gene transcription

**Figure 2:**
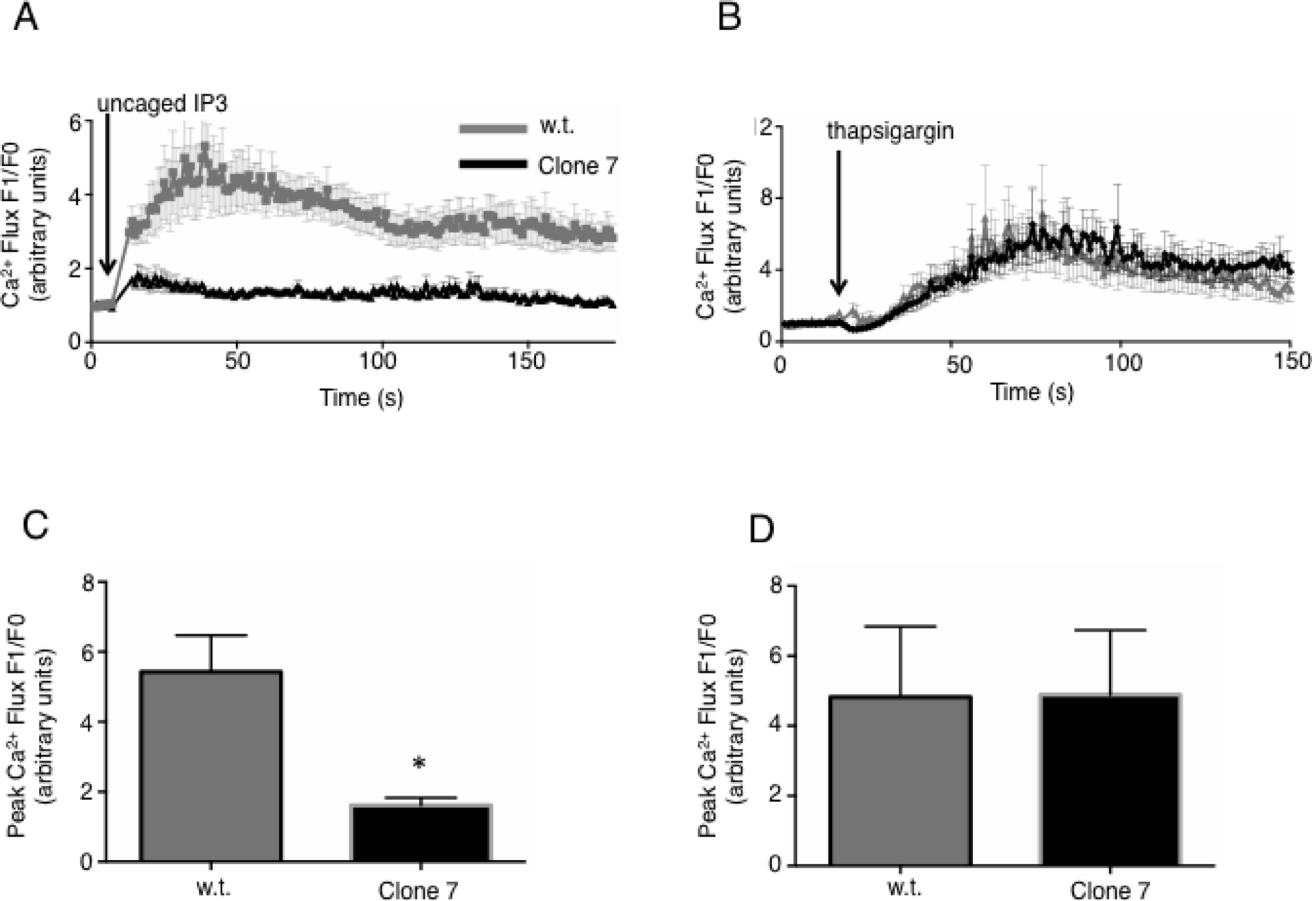
IP3R-dependent Ca^2+^ flux is reduced in SELENOK deficient melanoma cells (**A**) SK-MEL-28 w.t. and Clone 7 cells were loaded with both caged IP3 (0.5 µg/mL) and the Ca^2+^ sensitive fluorochrome, FuraRed. Confocal microscopy was used to measure baseline fluorescence for 30 sec, followed by exposure to a 2 sec *uv* light pulse to uncage the IP3 and measurement of fluorescence in response to IP3 binding the IP3R within the cells. Because FuraRed fluorescence decreases upon Ca^2+^ binding, the fluorescence intensity was inverted and plotted as signal after uncaging of IP3 (F1) divided by the average baseline signal (F0). Results showed reduced Ca^2+^ flux in Clone 7 cells. (**B**) Thapsigargin, which bypasses the IP3 receptor to induce a Ca^2+^ flux, was used to compare w.t. SK-MEL-28 to Clone 7 cells and no differences were found. Results for Ca^2+^ flux experiments represent three independent repeats that each included ≥30 cells per condition. (**C**–**D**) The above assays were repeated in 3 independent experiments, means of replicates were compared using a student’s *t*-test and expressed as mean + SEM with ^*^p < 0.05.

**Figure 3:**
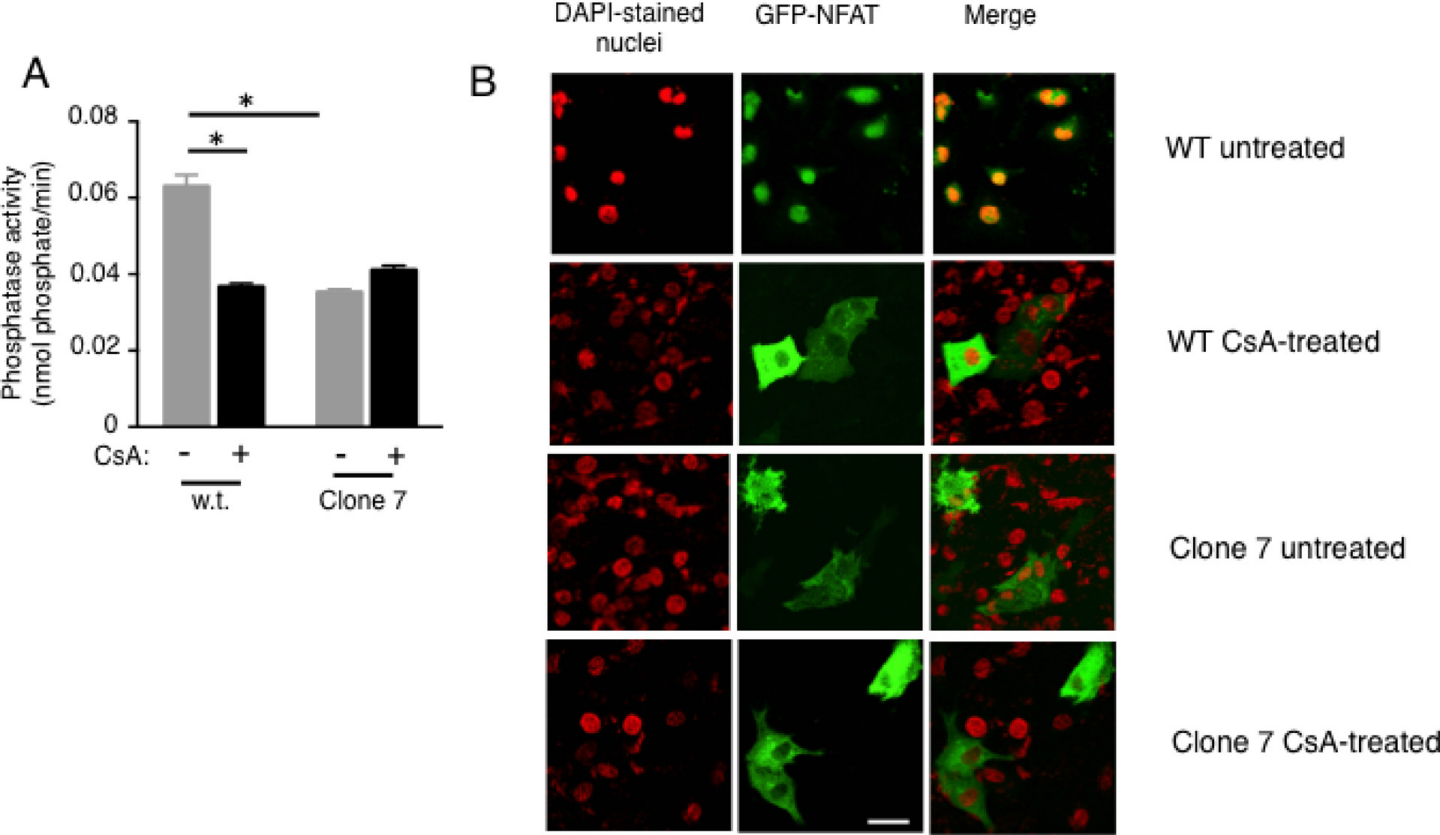
Calcineurin activity and NFAT nuclear localization are reduced in SELENOK deficient melanoma cells (**A**) Cells were incubated for 2 h with DMSO vehicle control or the calcineurin inhibitor, cyclosporin A (2 µM), and then cell lysates prepared normalized to total protein. The calcineurin substrate, RII phosphopeptide, was added to lysates and colorimetry used to detect production of phosphate. Means of replicates (*N* = 3) were compared using a student’s *t*-test and expressed as mean + SEM with ^*^p < 0.05. All experiments are representative of a minimum of two independent experiments. (**B**) Using the same cyclosporine A conditions, GFP-NFAT localization was evaluated using confocal microscopy and results showed that SELENOK deficient Clone 7 cells had accumulation NFAT in the cytosol similar to cells treated with Cyclosporin A. Note that DAPI staining of nuclei was recolored from blue to red to allow merged (yellow) to be more apparent. Scalebar represents 5 µm.

The reduced proliferation caused by SELENOK deficiency shown above may be due to reduced Ca^2+^ flux caused by the observed lower IP3R levels, similar to what has been found in SELENOK deficient immune cells [9]. To evaluate ability of the cells to generate Ca^2+^ flux, caged IP3 was loaded into w.t. cells and SELENOK-deficient Clone 7 cells and *uv* light was used to uncage the IP3 leading to IP3R-induced Ca^2+^ flux. Compared to w.t. controls, SELENOK-deficient Clone 7 cells exhibited reduced Ca^2+^ flux in response to uncaged IP3 (Figure 2A). Control experiments showing Ca^2+^ flux in response to the *uv* pulse alone (in the absence of uncaged IP3) are included in Supplementary Figure 3. In contrast to uncaged IP3, thapsigargin treatment bypasses the IP3R to induce Ca^2+^ flux, and this led to similar responses in Clone 7 cells compared to w.t. cells (Figure 2B). Peak Ca^2+^ flux from repeat experiments are shown in Figure 2C–2D. Insufficient Ca^2+^ flux in melanoma cells may interfere with calcineurin signaling [15], so we measured activity of this enzyme in w.t. and Clone 7 cells. Calcineurin activity in SELENOK deficient Clone 7 cells was lower compared to w.t. controls (Figure 3A). The calcineurin inhibitor, cyclosporin A, lowered w.t. levels to those exhibited in Clone 7 cells but cyclosporine A did not affect activity in Clone 7 cells. Importantly, differences in calcineurin activity was not do to differences in levels of calcineurin subunits A and B or calmodulin (Supplementary Figure 4). Activated calcineurin acts as phosphatase to cleave phosphate groups from the nuclear factor of activated T cells (NFAT) and induce translocation from the cytosol to the nucleus. We found higher levels of NFAT in the cytosol of Clone 7 cells compared to WT cells (Figure 3B), which is consistent with lower levels of endogenous calcineurin activity in SELENOK-deficient cells.

**Figure 4:**
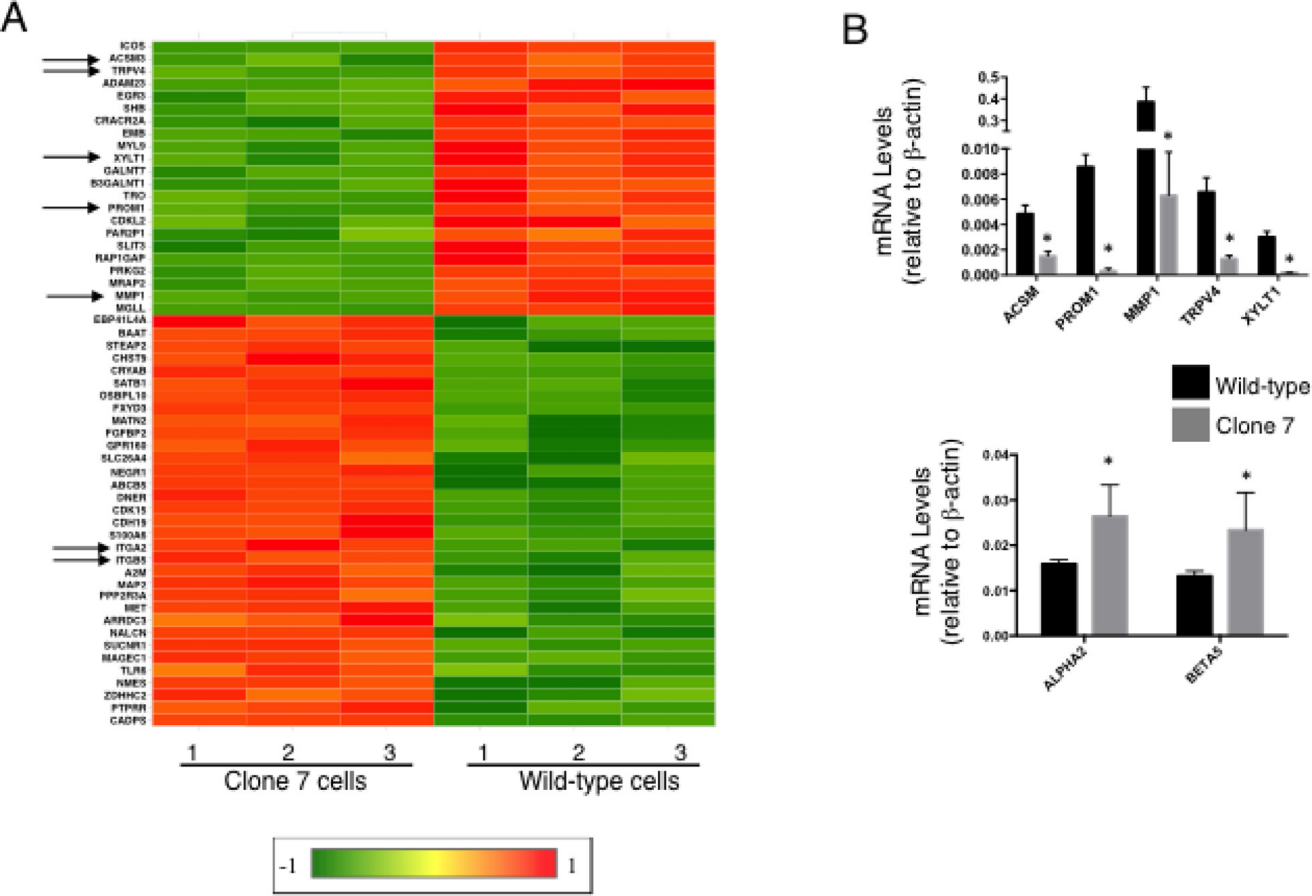
SELENOK deficiency alters transcriptional control of growth, migration and stemness (**A**) Gene arrays were used to compare SELENOK deficient Clone 7 cells to w.t. cells (*N* = 3 per group). Differentially expressed genes most related to cancer growth were selected based on Exp2 (Array Studio default) transformed > 2-fold higher or lower than controls with Benjamini & Hochberg FDR-adjusted *p*-value < 0.05. A heat map was generated using the linear gene expression values, which were normalized by dividing by w.t. average and robust center scaling with a linear range of 1 to -1. Down-regulated (green) and up-regulated (red) genes are shown for Clone 7 compared to w.t. cells or vice versa. Arrows indicate genes that were further evaluated. (**B**) Real-time PCR was used to measure relative mRNA levels for selected genes. Results confirmed that Clone 7 cells exhibited down-regulation of five transcripts involved in promoting growth, stemness, and migration (upper graph) and up-regulation of 2 transcripts involved in adhesion (lower graph). Means of replicates (*N* = 4) were compared using a student’s *t*-test and expressed as mean + SEM with ^*^p < 0.05.

**Figure 5:**
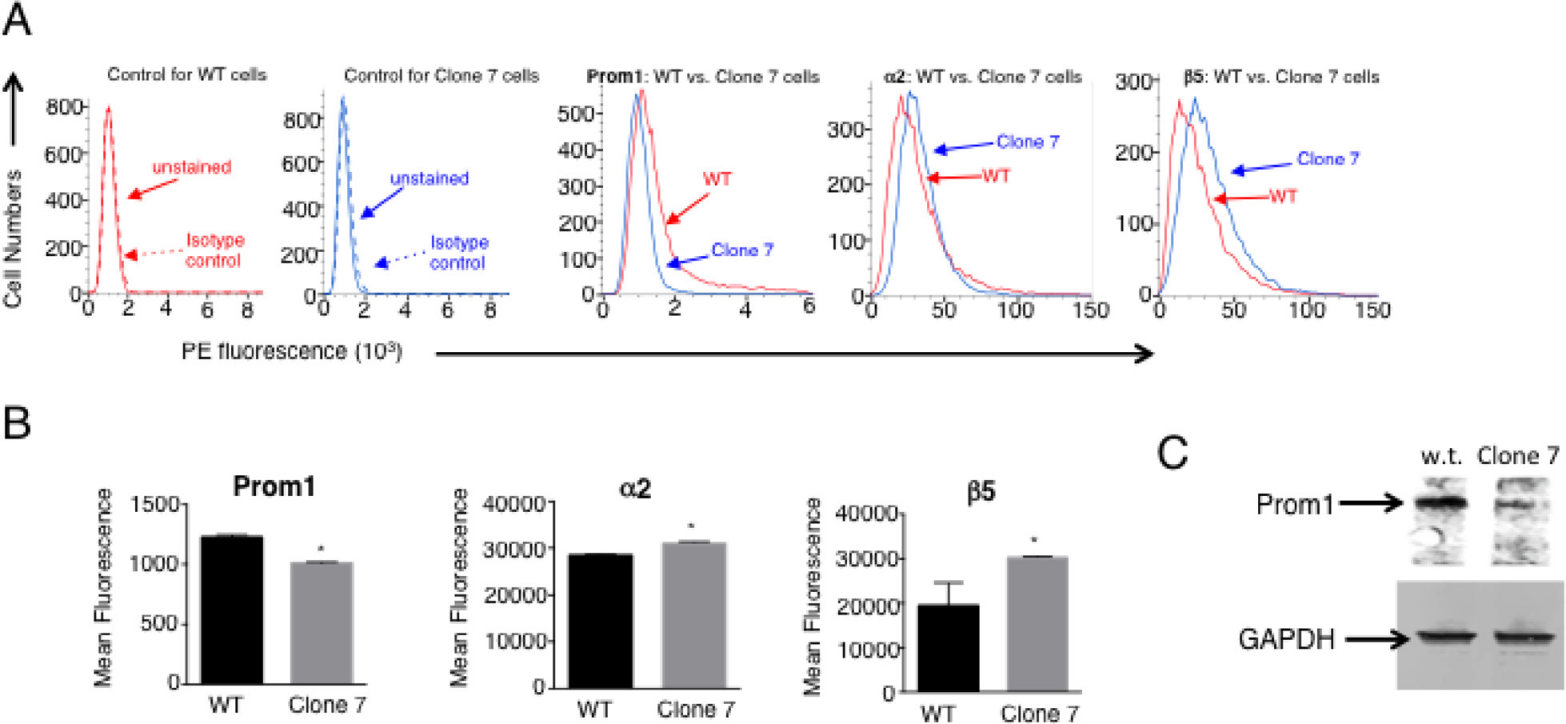
Protein levels for Prom1 as well as α2 and β5 integrin chains are altered similar to transcript levels with SELENOK deficiency (**A**) Flow cytometry was used to evaluate surface expression of Prom1 along with α2 and β5 integrin chains. PE-labeled isotype control antibodies did not bind either wild-type (WT) cells or Clone 7 cells (two left panels). For Clone 7 cells, fluorescence intensity decreased for Prom1 and increased for α2 and β5 integrin chains. (**B**) The fluorescence intensity was measured for replicates (*N* = 3) and means were compared using a student’s *t*-test and expressed as mean + SEM with ^*^p < 0.05. (**C**) Prom1 protein levels were also evaluated using western blot with GAPDH used as a loading control.

These results suggested that SELENOK deficiency affects calcineurin that may lead to altered gene transcription, so gene array experiments were conducted that led to identification of 166 up-regulated and 113 down-regulated transcripts by > 2-fold in Clone 7 cells compared to w.t. controls. A full list of transcripts significantly up- or down- regulated by > 2-fold is shown in Supplementary Figure 5 and a heat map was generated including genes related to cancer properties (Figure 4A). Transcripts decreased by SELENOK-deficiency included some involved in promoting growth and metabolism (e.g. ACSM3 and XYLT1), invasion and metastasis (e.g. MMP1 and TRPV4), and stemness (e.g. PROM1). Interestingly, SELENOK-deficiency increased levels of transcripts involved in promoting cellular attachment (e.g. ITGA2 and ITGB5). Real-time PCR confirmed changes in levels of these transcripts (Figure 4B). Protein levels for these factors were evaluated using flow cytometry and western blot. Consistent with the changes in mRNA, protein levels for PROM1 were decreased and α2 and β5 integrin chains were increased by SELENOK deficiency (Figure 5A–5C). Signaling pathway analyses revealed how these factors and other signaling nodes that impinge upon cancer related functions were affected by the lack of functional SELENOK (Supplementary Figure 6). Overall, SELENOK deficiency in melanoma cells led to reduced capacity for IP3R-generated Ca^2+^ flux and altered gene transcription in a manner that disrupted signaling programs involved cellular growth and stemness as well as migration and attachment.

### Cellular migration and attachment are altered with SELENOK deficiency

**Figure 6:**
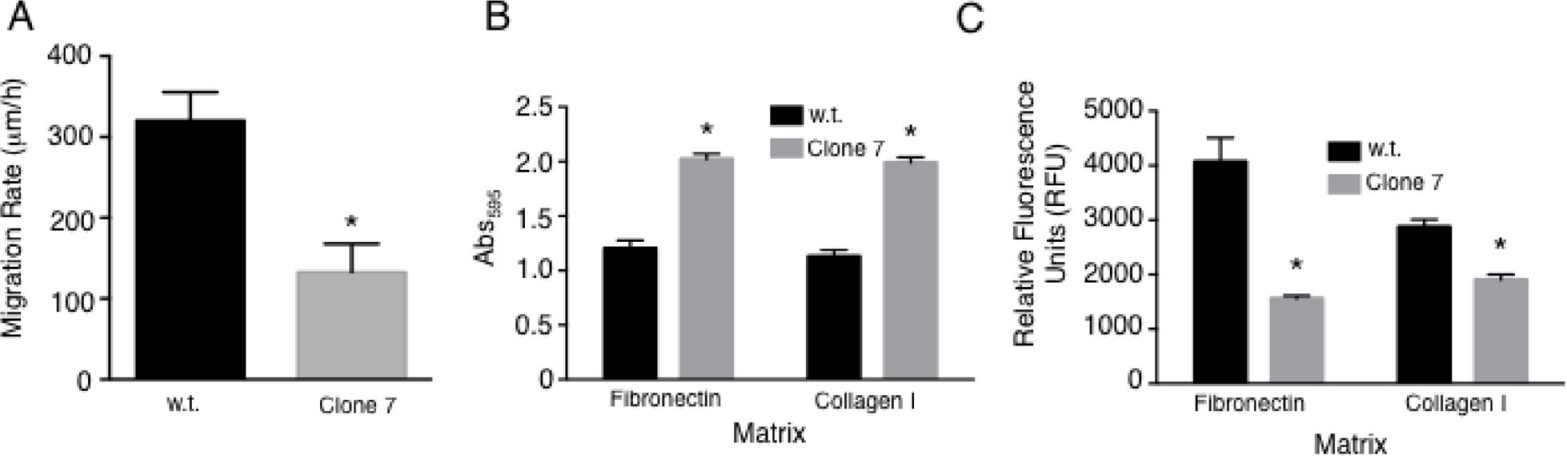
SELENOK deficiency alters adhesion and migration of melanoma cells (**A**) Migration rates were measured for w.t. SK-MEL-28 cells and SELENOK deficient Clone 7 cells using electrode-created wounds and resistance to measure movement of cells into cleared electrode areas. (**B**) Adherence to fibronectin and collagen I was measured and Clone 7 cells found to exhibit increased adherence to both types of fibers compared to w.t. controls. Stained cells were measured using absorbance read on an ELISA plate reader at 595 nm. (**C**) Transwell assays were performed to test w.t. and Clone 7 cells for migration through fibronectin or collagen I. Migration through both matrices was lower for Clone 7 cells compare to w.t. controls as measured by fluorescent staining of cells. Results represent three independent experiments. Means of replicates, *N* = 3 for (A) and *N* = 6 for (C–**D**), were compared using a student’s *t*-test and expressed as mean + SEM with ^*^p < 0.05.

The increases in mRNA and protein levels shown above for integrin chains were modest, but suggested that cellular attachment and migration may be affected. To assess the rate of migration, an electrode/resistance assay was used to measure cell movement over time for SELENOK deficient Clone 7 compared to w.t. SK-MEL-28 cells. Results showed that the Clone 7 cells were impaired for migration compared to w.t. controls (Figure 6A). Extracellular matrix proteins play an important role in the initial steps of invasion and migration during metastasis, so we next performed experiments with fibronectin or collagen I matrices including adherence and transwell migration assays. SELENOK deficient Clone 7 cells showed higher levels of adherence and lower transwell migration in the presence of both fibronectin and collagen I compared to w.t. cells (Figure 6B–6C). These results suggest that deficiency in functional SELENOK in melanoma cells increases adhesion to extracellular matrix while reducing their capacity to migrate.

### *In vivo* SELENOK-deficiency reduces melanoma progression and metastasis

**Figure 7:**
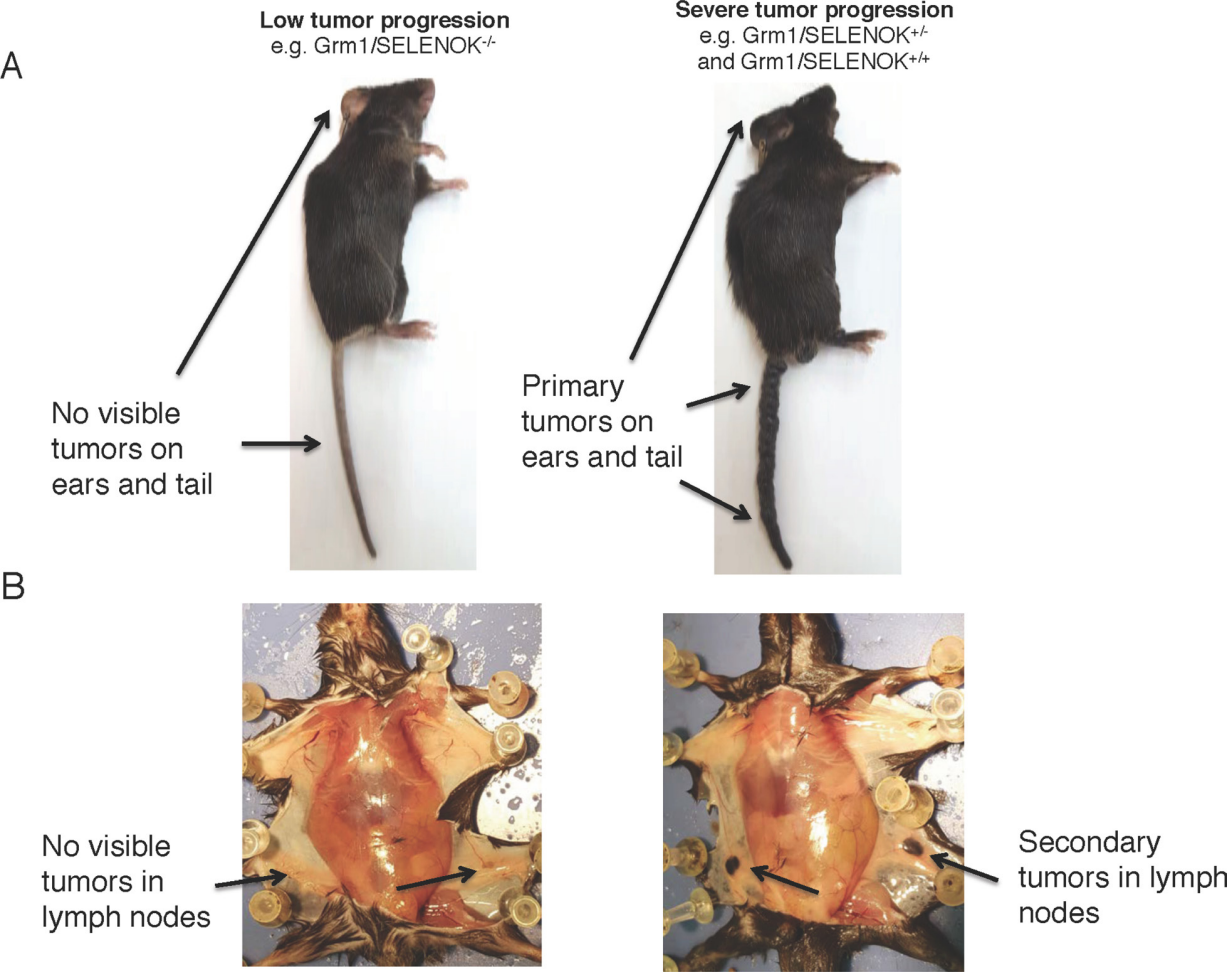
Deletion of SELENOK *in vivo* leads to reduced primary and secondary tumors in Grm1-Tg mice as analyzed by gross anatomy The Grm1-Tg mouse line spontaneously develops tumors on tails and ears that migrate to draining lymph nodes by 4 months of age. These mice were bred with SELENOK knockout mice to generate littermates: Grm1/SELENOK^+/+^, Grm1/SELENOK^+/–^, and Grm1/SELENOK^–/–^ mice. Upon evaluation of 4-month-old mice it was found that some mice exhibited negligible tumor progression while others exhibited high levels of primary (**A**) and secondary (**B**) tumors. Cross referencing of genotypes with gross anatomy revealed that the mice with very low tumor formation were all of the Grm1/SELENOK^–/–^ genotype, while all of the Grm1/SELENOK^+/+^ and Grm1/SELENOK^+/–^ ranged from intermediate to high tumor growth.

To evaluate the importance of SELENOK for progression of melanoma *in vivo*, we utilized a spontaneous melanoma transgenic (Tg) mouse model involving overexpression of the glutamate receptor 1 (Grm1) under control of the melanocyte specific tyrosine-related protein 2 (Trp2) promoter [16, 17]. This established model is characterized by the development of melanoma in the ears and tails of newborn mice, which progresses to metastatic melanoma with cancer cells appearing in the draining lymph nodes by 4 months of age and 100% penetrance. Using these mice, we carried out a breeding scheme to delete SELENOK gene on the Grm1-Tg background. This generated littermate controlled experiments consisting of Grm1/SELENOK^+/+^, Grm1/SELENOK^+/–^, and Grm1/SELENOK^–/–^ mice. This allowed us to compare the littermates for the development of primary and secondary tumors. Male and female cohorts from these litters were analyzed separately for tumors on tails and ears at 4 months of age. Gross anatomical evaluation of littermates revealed that Grm1-Tg mice having both SELENOK alleles knocked out exhibited dramatically lower levels of primary and secondary tumors (Figure 7). Those Grm1-Tg mice with either one or two intact SELENOK alleles developed intermediate to severe melanoma on tails and ears with the appearance of pigmented cells in the draining lymph nodes. The results were similar for males and females, suggesting no dimorphic effects of SELENOK deficiency on melanoma in this model. The melanoma in this mouse model is not lethal [17], which limits the ability to perform survival analyses. However, the mice in our experiments were followed up to 8 months of age and the Grm1/SELENOK^–/–^ mice continued to exhibit lower levels of tumor formation compared to Grm1/SELENOK^+/+^ and Grm1/SELENOK^+/–^ littermates for both sexes (Supplementary Video 1).

**Figure 8:**
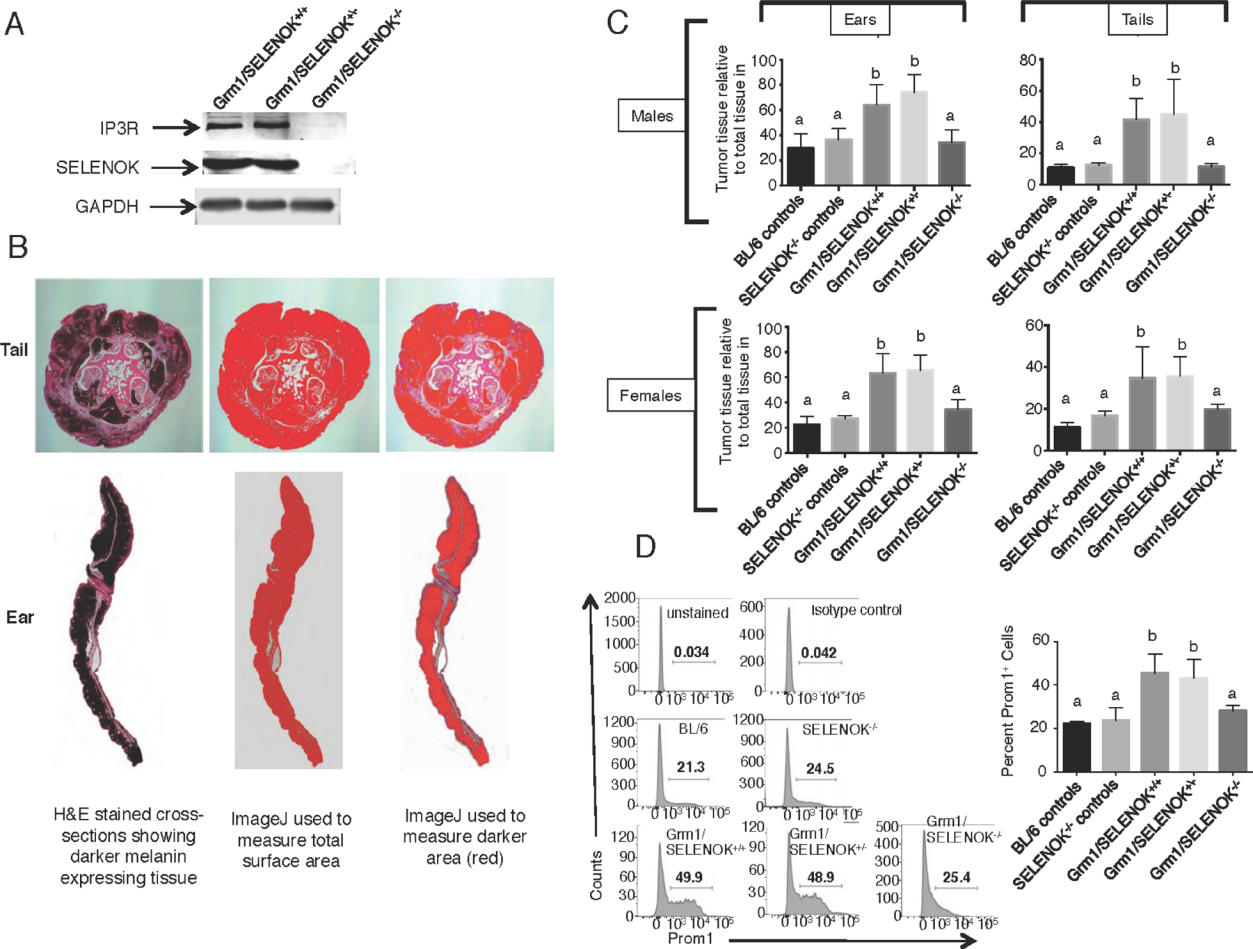
SELENOK deficiency reduces melanoma tumor growth on tails and ears (**A**) Western blot analysis of splenocytes, which express relatively high levels of SELENOK and IP3R, confirm that complete knockout of SELENOK leads to reduced IP3R in Grm1/SELENOK^–/–^ mice. (**B**) Male and female littermates at 4 months of age with genotypes of Grm1/SELENOK^+/+^, Grm1/SELENOK^+/–^, and Grm1/SELENOK^–/–^ were compared for levels of melanin positive tissue relative to total tissue in tails and ears. (**C**) Cross-sections of tails and ears were H&E stained and evaluated using an ImageJ color threshold approach as described in the Methods section. The dark melanin area relative to total area of tissue was compared for all three littermate genotypes. We also included two control groups expected to form no tumors (C57BL/6 and SELENOK^–/–^) due to a lack of the Grm1 transgene in melanocytes, which allowed assessment of melanin content in the absence of melanoma. Results showed that Grm1/SELENOK^–/–^ mice had significantly reduced tumor growth in tails and ears, while Grm1/SELENOK^+/+^ and Grm1/SELENOK^+/–^ mice were similar in progression of melanoma. Similar results were found with males and females. (**D**) Cells dissociated from were analyzed by flow cytometry for Prom1^+^ cancer stem cells and lower levels were found in Grm1/SELENOK^–/–^ mice compared to Grm1/SELENOK^+/+^ and Grm1/SELENOK^+/–^ littermates. Both sexes were included in each group, with representative images shown on the left and data graphed on the left (*N* = 3). A one-way ANOVA with Tukey post-test was used to analyze groups and means without a common letter differ, *p* < 0.05.

We evaluated these mice for affects of SELENOK deficiency on IP3R and, as expected, the littermates with a complete deletion of SELENOK exhibited lower IP3R levels (Figure 8A). To quantify tumor tissue in tails and ears, a microscopy approach was taken involving color threshold analyses of melanin levels in tail and ear tissue cross-sections. The three littermate genotypes, Grm1/SELENOK^+/+^, Grm1/SELENOK^+/–^, and Grm1/SELENOK^–/–^, were analyzed for both male and female mice at 4 months of age (*N* = 7 per sex per genotype). We also included two control groups expected to develop no tumors due to lack of the Grm1 transgene: C57BL/6J and SELENOK^–/–^ mice. The results clearly showed that Grm1-Tg mice lacking SELENOK expression had very low levels of pigmented cells in ears and tails (Figure 8B–8C). In fact, the levels of pigmented tissue for Grm1/SELENOK^–/–^ mice were similar to the C57BL/6J and SELENOK^–/–^ control mice lacking the Grm1 transgene. Cells were dissociated from tails and flow cytometry used to analyze levels of cells expressing the Prom1 cancer stem cell marker [18, 19]. Results showed that Grm1/SELENOK^–/–^ mice exhibited lower levels of these cells (Figure 8D), which is consistent with the cell line data described above showing that SELENOK deficiency decreases stemness.

**Figure 9:**
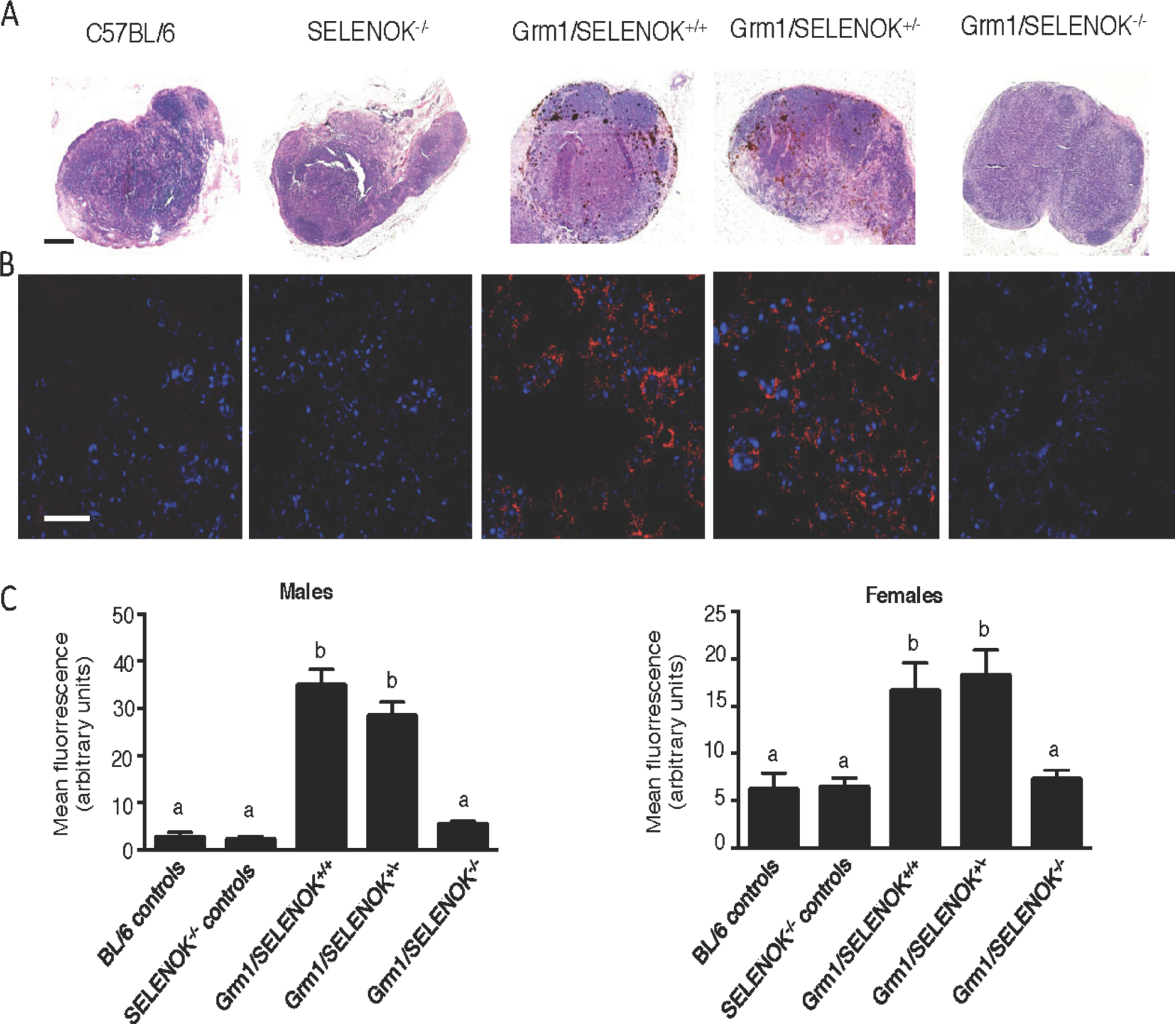
SELENOK deficiency reduces metastasis of melanoma to lymph nodes Male and female littermates at 4 months of age with genotypes of Grm1/SELENOK^+/+^, Grm1/SELENOK^+/–^, and Grm1/SELENOK^–/–^ were analyzed for presence of melanoma cells in inguinal and axillary lymph nodes (*N* = 7/group). Non-tumor mice were included as controls (*N* = 3/group). (**A**) H&E stained lymph node sections were examined under low power (5×) for melanin, which appears as dark brown cells. Scalebar = 2 mm. (**B**) Immunofluorescence (40×) was used to detect the melanin specific antigen, Trp2 (red), in lymph node sections. DAPI (blue) was used to stain nuclei. Scalebar = 20 mm. (**C**) Trp-2 fluorescence was quantified using ImageJ with a minimum of 7 mice per group and 2 sections analyzed per mouse. A one-way ANOVA with Tukey post-test was used to analyze groups and means without a common letter differ, *p* < 0.05.

To evaluate metastasis in these mice, the draining lymph nodes (inguinal and axillary) were evaluated by two approaches. First, light microscopic evaluation of melanin in the lymph nodes was used for qualitative assessment (Figure 9A). Second, immunofluorescence detection of the melanocyte specific antigen, Trp2, was quantified (Figure 9B–9C). The latter was included due to a report that melanoma cells that have migrated to secondary tissues in this model may lose some melanin expression [16]. Specificity of this immunofluorescence approach was confirmed (Supplementary Figure 7). Similar to the primary tumor data described above, results showed significantly reduced melanoma in the lymph nodes of Grm1/SELENOK^–/–^ mice compared to Grm1/SELENOK^+/–^ and Grm1/SELENOK^+/+^ mice, which were similar to each other. Also consistent with primary tumor data, SELENOK deficiency in the Grm1-Tg mice led to a reduction of Trp2-positive cells in the lymph nodes at levels similar to mice lacking tumors (C57BL/6J and SELENOK^–/–^ controls). Overall, these data suggest that SELENOK expression is required for progression of primary melanoma tumors as well as metastasis to the draining lymph nodes.

## DISCUSSION

To date, a role for SELENOK in cancer progression has not been described and the data presented herein suggest that this selenoprotein acts to promote melanoma tumor progression and metastasis. Our results suggest that this is related to its role in promoting stable expression of the IP3R in the ER membrane, which is required for efficient Ca^2+^ flux and downstream Ca^2+^-dependent signaling events that promote growth, migration, and stemness. A number of studies have shown the importance of SELENOK in maintaining IP3R-dependent Ca^2+^ flux in immune cells [9, 20, 21], but the data presented herein demonstrate a new role in promoting Ca^2+^-dependent functions such as proliferation and migration in cancer cells both *in vitro* and *in vivo*.

The importance of Ca^2+^ flux in regulating melanoma proliferation and cell migration was previously suggested by experiments including a knockdown of STIM1 or Orai1 in human melanoma cell lines as well as a pharmacological inhibitor of SOCE [22]. Similar effects were shown for breast tumor cells and glioblastoma cells [23, 24]. Our data reveal that calcineurin activity is reduced in melanoma cells, and this can lead to loss-of-function (growth, migration) as well as gain-of-function (attachment to matrix) that may perturb *in vivo* disease progression. Substantial Ca^2+^ flux gain-of-function hindering survival and progression of cancers cells has also been shown by studies in which sodium butyrate, a histone deacetylase inhibitor, induced apoptosis in nasopharyngeal carcinoma cells by enhancing SOCE [25]. This notion of optimal levels of Ca^2+^ flux being important was supported by a study in which overexpression of Orai1 in A459 lung cancer cells inhibited EGF-mediated cell proliferation [26]. Ca^2+^ levels in malignant melanoma regulate a number of downstream signaling pathways such as S100B binding to p90 ribosomal S6 kinase that increases cell survival [27]. Thus, the timing and levels of Ca^2+^ flux from the ER stores must be tightly controlled and this further supports the notion that disruption of Ca^2+^ flux regulation may serve as a therapeutic target for treating melanoma and other cancers.

SOCE has been described in the context of cancer, including a role for a switch in Ca^2+^ entry from SOCE-dependent to -independent phenotypes as an oncogenic driver [28, 29]. Our gene array data suggest that growing melanoma cells require SELENOK for proliferation and stemness transcriptional programs to modify their phenotype, we cannot categorical state that this is due solely on SOCE. Interestingly, Stim and Orai transcripts were not included in those mRNAs affected by SELENOK deficiency. Also, we tried several experiments to implicate Ca^2+^ flux using CsA or FK506 inhibitors on w.t. and Clone 7 cells followed by real-time PCR measuring levels of the mRNAs found to be affected by SELENOK deficiency. We included several time periods to determine how long the inhibitors took to exert effects on transcription. However, we were not able to maintain consistent results using the CsA and FK506, and it may be technical issues or the non-synchronized nature of the growing cells that led to inconclusive results. Thus, our results support a role for SELENOK in transcriptional programs supporting melanoma growth and stemness, but we cannot conclude that transcriptional changes are directly due to the effects of SELENOK on Ca^2+^ flux that causes the changes in gene expression levels. It is also worth noting that a lower activity of calcineurin caused by SELENOK deficiency leading to lower NFAT nuclear localization could reduce levels of some transcripts, but it is less intuitive how that may lead to higher mRNA levels for other genes. It could be that these increases are indirect and result as secondary effects arising from lower NFAT in the nucleus causing other transcription factors to increase in their activity.

Metastasis involves a loss of cell adhesion from the primary tumor, cell migration through extracellular matrix, adhesion to a secondary site then cell proliferation. These steps rely on tightly regulated Ca^2+^ flux [30], and our data show that the loss of SELENOK expression in SK-Mel-28 cells led to increased adhesion and decreased migration. Interestingly, only one intact SELENOK allele was required for *in vitro* melanoma cell functions and *in vivo* tumor growth. This suggests a high level of SELENOK protein is not necessarily required for malignant melanoma to progress. This is consistent with our data in human tissues and NCI-60 melanoma cell lines showing that SELENOK protein levels are not increased in melanoma compared to normal cells or tissues. While changes in the expression and function of STIM1 and Orai1 have been found in a range of cancer types and thus implicated in disease progression, levels of STIM1 and Orai1 were not different between malignant and non-malignant melanoma cells [31]. In fact, there remains heterogeneity in levels of these and other SOCE proteins (e.g. IP3Rs) in melanoma [14]. Since SELENOK is a cofactor that binds to the DHHC6 enzyme to catalyze the palmitoylation of the IP3R required for its stability in the ER membrane, perhaps only small amounts are required and an increase in SELENOK may not lead to more IP3R assembled as a Ca^2+^ in the ER membrane. However, genetic manipulation of the SELENOK gene *in vitro* and *in vivo* leading to substantially lower levels of full-length protein clearly decreased SOCE in melanoma cells. This suggests a threshold of SELENOK exists that is needed for efficient Ca^2+^ flux in melanoma cells, similar to what has been observed for immune cells [9].

Targeting SOCE and/or Ca^2+^ signaling for cancer therapy is an emerging interest in cancer research [7]. STIMs and ORAIs have emerged as possible molecular targets for cancer therapeutics [32]. However, Ca^2+^ flux plays a crucial role in non-cancer cells for survival, metabolism, differentiation and movement [33]. In addition, specific calcium signalling pathways have also now been identified as playing important roles in the establishment and maintenance of multidrug resistance and the tumour microenvironment [34]. Homozygous mutations in or targeted disruption of STIM-1 or Orai1 results in perinatal lethality, while STIM-2 knockout mice only survive to ∼5 wks of age [35–38]. The fact that the SELENOK knockout mice show very little phenotypic defects and are completely fertile suggests that therapeutic targeting of this factor may generate far less side effects. This notion combined with the impressive reduction of melanoma with SELENOK in the mouse model of melanoma presented herein support further investigation of this protein for treatment of melanoma or other metastatic cancers. Genetic interruption of the gene encoding SELENOK eliminates expression of SELENOK from gestation and this is obviously not equivalent to using inhibitor drugs, which would interfere with the function of this factor only upon delivery of the drug. This is a limitation in our genetic approach in totally knocking out SELENOK and we are planning studies involving inducible knockdown of SELENOK that would better reflect therapeutic intervention approaches to reduce melanoma progression. However, our previous work identified the functional domain of SELENOK consisting of amino acids 82–94 [11, 13], which may guide future development of small molecule inhibitors. Overall, the *in vitro* and *in vivo* data regarding SELENOK deficiency and melanoma support further investigation into this protein as a potential therapeutic target.

## MATERIALS AND METHODS

### Mice

The generation of SELENOK^–/–^ mice have been previously described [9], and C57BL/6J obtained from Jackson Laboratories were used as controls. Grm1-transgenic (Tg) mice [16, 17] were obtained from Dr. Suzie Chen at Rutgers University and used to establish a transgenic colony. Animal protocols were approved by the University of Hawaii Institutional Animal Care and Use Committee.

### Cells, antibodies and reagents

Lysates from primary melanocytes were purchased from Sciencell Research Laboratories (Carlsbad, CA) and NCI-60 validated human melanoma cell lines obtained from the University of Hawaii Cancer Center included SK-Mel2, SK-Mel28, and MALME-3M. These cell lines were cultured in RPMI media with 10% fetal bovine serum and 1% Antibiotic-Antimycotic (all from GIBCO/Thermo Fisher). Primary antibodies for western blots included rabbit monoclonal anti-SELENOK (Epigemonics, Inc., custom antibody), anti-IP3R1 (Santa Cruz Biotechnology, sc-271197), anti-GAPDH (Santa Cruz Biotechnology, sc-47724), anti-Prom1 (Cell Signaling, 58605). Antibodies from Abcam included anti-TRP2 (ab74073), anti-calcineurin A and B (ab137335, ab154650), and anti-calmodulin (ab 105498, respectively). Western blot secondary antibodies were purchased from Li-Cor Technologies and immunofluorescence secondary Alexafluor595 antibody from Thermo. The transgene encoding full-length SELENOK in pcDNA3.1+ (Invitrogen) has been previously described [11].

### Proliferation and migration assays

To measure proliferation, cells were grown to 90% confluency in flasks and then trypsinized, resuspended and plated at 4000 cells per well in 4 separate black 96-well plates. On each day 1, 2, 3, and 4 one plate was removed from incubation and centrifuged at 400 RCF for 4 min to pellet cells, the media removed and plates wrapped in parafilm and stored at –80° C. On day 5, pellets were thawed and lysed and cells quantified using a CyQUANT^®^ Kit (ThermoFisher, Inc.). A fluorescence plate reader (Molecular Devices, Inc.) was used to quantify fluorescence of samples and compare to a standard curve generated using SK-Mel-28 cells quantified using a Millipore Sceptor (Life Sciences, Inc.). Scratch assays were performed as previously described [39]. Transwell migration assays were carried out as previously described [40]. Lower sides of transwell filters (8.0 µm pore size; Greiner Bio-One) were coated with 15 µg/mL human plasma fibronectin or collagen I overnight at 4° C. Cells (10^6^ cells/mL) were plated on the top side of the filters in serum free media and incubated for 20 h at 37° C and 5% CO_2_. The number of cells that migrated through the membrane was measured by Calcein-AM dye (Sigma-Aldrich) uptake and fluorescence analyzed at 495 nm excitation/515 nm emission on a plate reader.

For measuring migration rates, cells grown to 80% confluency were trypsinized (0.05% trypsin/EDTA), washed and seeded in fresh media onto ECIS 8W10E+ PET slides at a concentration of 2.5 × 10^5^ cells/mL. Migration was measured using a wound-healing assay on an 1600R ECIS instrument (Applied Biophysics, Inc.) as described previously [41]. The rate of migration was measured as a function of time needed to climb back up to base resistance levels. Migration rate = electrode radius (µm)/ ∂time (h); ∂time = t_2_–t_1_ where t_2_ = time point in hours when resistance reading reaches base reading, t_1_ = time point in hours when cells were wounded.

### Adhesion assay

The cell attachment assay has been previously described [42]. Briefly, microtiter plates coated overnight at 4° C with human plasma fibronectin (Millipore) or collagen I (Upstate NY) at 10 µg/mL were blocked with 3% BSA for 1 h at 37° C, wells were rinsed once with PBS (pH 7.4) and cells were added (10^5^ cells/mL) in serum free media. Cells were incubated for 60 min at 37° C and 5% CO_2_. After 60 min, plates were washed with PBS, fixed with 3% paraformaldehyde, stained with 0.5% crystal violet/20% MEOH, washed under running distilled water, solubilized in 0.5% Triton X-100 in distilled water overnight at room temperature and absorbance read on an ELISA plate reader at 595 nm.

### Flow cytometry and western blots

Flow cytometry was performed on 5 × 10^5^ cells by incubating with phycoerythrin (PE)-conjugated isotype control IgG or IgG specific for Prom1 or α2 or β5 (all from BioLegend) used at 5 µL per test as recommended by vendor. Dead cells were excluded from analyses using violet blue exclusion dye (BioLegend). Samples were analyzed using the Attune NxT flow cytometer (ThermoFisher) and analyzed using FlowJo software. Epidermal cells were dissociated from tails using a GentleMacs and Epidermis Dissociation Kit (Miltenyi). Cells were incubate with Fc-block followed by APC-anti-Prom1 (both BioLegend per vendor’s instructions), cells fixed with 2% paraformaldehyde and analyzed on a Fortessa flow cytometer (BD Biosciences). Western blotting methods were followed as described previously [43], and bands were visualized using a Li-Cor Odyssey.

### Genome editing and transfections

A CRISPR/Cas9 approach was used to mutate the SELENOK gene (NCBI GeneID 58515) in order to generate a cell line expressing a truncated, nonfunctional SELENOK protein. First, SK-Mel28 cells were transfected with a pSpCase9(BB)-2A-GFP (Addgene) using Lipofectamine 3000 (ThermoFisher). After 24 h, GFP-positive cells were sorted using a FACSaria (BD Bioscience) into medium with 20% FBS without antibiotics. These cells were transfected with Integrated DNA Technologies (IDT) guide RNA and transactivating RNA complexes premixed in IDT complex buffer for 30 min using Lipofectamine 3000 per IDT recommendations. Cells were recovered in medium with 20% FBS without antibiotics for 24 hours, then 7-AAD Viability Stain Solution (Biolegend, Inc.) was added and another round of cell sorting carried out using the FACSaria instrument. The GFP^+^7AAD^-^ cells were sorted into 96-well plate with one cell per well in 200 mL of medium with 20% FBS and no antibiotics. After ∼2.5 weeks, live clones were transfered to 24-well plates and further grown into colonies. Colonies were screened by western blot for truncated SELENOK protein. Those clones exhibiting desired changes in SELENOK levels or size were confirmed as edited by Sanger sequencing.

### Ca^2+^ flux, calcineurin activity assays, and NFAT localization

Ca^2+^ flux was assayed using a cell permeable, caged IP3 molecule as previously described [12], by loading cells with a HBSS/1%FBS solution containing 0.5 µg/mL caged IP3 (Enzo Life Sciences) and FuraRed/Pluronic acid (Life Sciences) for 30 min at 37° C. The media was replaced with phenol red free RPMI containing 5% FBS to allow the cells to recover for at least 1 h. The coverslips were then mounted onto RC-25F chambers (Warner) and placed into the mount of a Zeiss Pascal inverted confocal microscope with LSM5 dual lasers and camera. The fluorescence intensity of the FuraRed was measured at 2 sec intervals for 1 min and then cells exposed to *uv* light for 5 sec to uncage the IP3 molecule and fluorescence measurements continued for 3 min. The thapsigargin was used as a control as previously described [9]. The ratio of red fluorescence was inverted to represent increased Ca^2+^ since FuraRed signal decreases with increased cytosolic Ca^2+^ and results expressed as F1/F0. Phosphatase activity was measured on lysates prepared from SK-MEL-28 cells and Clone 7 cells using the calcineurin cellular activity assay kit (Enzo). Cells were treated for 2 h with either 2 µM cyclosporine A (Sigma) in DMSO or DMSO as a vehicle control and lysates were normalized to equal total protein as determined by Bradford assay. Phosphatase activity was measured from cellular protein extracts as the dephosphorylation rate of a calcineurin specific phosphopeptide substrate (R-II phosphopeptide) in the presence of 0.5 mM CaCl_2_/0.25 μM calmodulin. The colorimetric assay was measured on a SpectraMax 384 microplate reader (Molecular Devices, Inc.). NFAT localization was assessed by transfecting cells with a plasmid encoding GFP-c1NFAT3 (Addgene) using Lipofectamine 3000 (Invitrogene) with or without 2 µM cyclosporine A (Sigma) for 18 h. Immunofluorescence microscopy was carried out with a Leica Microsytems LAS AF TCS SP5 confocal microscope to assess cytoplasmic versus nuclear localization.

### Microarrays

SK-MEL-28 w.t. cells and Clone 7 cells were harvested at 80% confluency were plated at in 6-well plates (6 × 10^5^ cells per well) and grown for 48 h followed by RNA isolation using the EZNA Total RNA kit (Omega Biotek, Inc). RNA integrity was valdated on Agilent 2100 Bioanalyzer using RNA Nano chip. For gene expression profiling, 100 ng of total RNA was used for downstream processing using GeneChip Whole Transcript Expression protocol followed by hybridization to Clariom S, Human Arrays (Affymetrix, Santa Clara, CA, USA). Subsequently, arrays were washed, stained and scanned using GeneChip Fluidics Station 450 and GeneChip Scanner (Affymetrix). Generated CEL files were normalized using the SST-RMA-GENE-FULL algorithm in the Affymetrix GeneChip Expression console software. Raw CEL files generated from Clarion S human microarray were preprocessed in Array Studio (version 9; OmicSoft, Cary, NC) using data normalization by the Robust Multi-array Average (RMA) approach. Genes with Exp2 (default) transformed fold change greater or less than 2 and Benjamine & Hochberg FDR-adjusted *p*-value < 0.05 were considered as differentially expressed in KO with respect to WT.

### Histological and immunofluorescent analyses of tissues

Tails and ears from 4-month-old mice were removed in an equivalent manner from each mouse and weighed. The ears were fixed in 10% buffered formalin for 1 wk. To ensure the same areas of tails were analyzed for tumors for each mouse, a cross-section was excised 20 mm from the base that was further cross-sectioned into 4 pieces 1 mm in length. All four tail sections were mounted in the same orientation in tissue paper and fixed for 1 wk in 10% buffered formalin. These fixed samples were then incubated in Trilogy™ Tris/EDTA (ThermoFisher) to remove cartilage for 1 wk. The ear and tail samples were dehydrated in a series of ethanol solutions and embedded in paraffin, sectioned and stained using a Leica CM1900 UV cryosectioner, Leica EG1160 and Microm HM340E microtomes, and a Leica automated tissue processor followed by standard Hemotoxylin and Eosin (H&E) staining of tissues. The inguinal and axillary lymph nodes were processed for both immunohistological and immunofluorescence staining. Processing for H&E staining followed the protocol described above. For immunofluorescent staining, sectioned samples on glass slides were dried overnight at RT and deparaffinized and rehydrated under high temperature and pressure to unmask antigens. ImmEdge pen was used to create hydrophobic border around the tissue and 5% goat serum (Vectastain) added for 1 h to block non-specific binding. Primary antibody (rabbit anti-Trp2; Abcam, Inc.) was added at a final concentration of 1:500 in 5% goat serum in PBS overnight at 4° C. After three washes with PBS, secondary antibody (rabbit Alexafluor594; ThermoFisher) was added at a final concentration of 1:1000 in 5% goat serum in PBS for 1 h at RT. After three washes with PBS, tissues were mounted in vectashield H-1200 with DAPI (Vector Labs, Inc.).

The H&E stained sections were imaged using a Zeiss Axioskop 2 Plus upright light microscope with camera and AxioVs40 V 4.7.2.0 acquisition software, equipped with a 5X Plan neofluor objective, 5X/.15, α/.17 numerical aperture of 0.9. Light microscope images were analyzed using ImageJ software. The amount of tumor tissue present in ears and tails were determined by setting the signal intensity representing dark melanin at threshold and then measuring the total surface area of tissue along with the surface area above the threshold. The percentage of melanin tissue above color threshold per total surface area was calculated. The lymph nodes were analyzed by immunofluorescence using a Leica Microsytems LAS AF TCS SP5 confocal microscope with a 40x oil objective with 5x zoom, HCX PL APO CS with numerical aperture of 1.3. The Laser lines used were 405 nm (for DAPI) and 594 nm (for Alexafluor594). Images were captured using LAS AF version 2.7.3.9723 acquisition software, 1997–2012 LeicaMicrosystems CMS GmbH. A minimum of 5 fields per sample was captured and ImageJ was used to analyze tissue for Trp2 signal with data presented as mean fluorescence intensity.

### Statistical analyses

Comparison of two means was carried out using an unpaired Student’s *t* test using GraphPad Prism version 4.0. In assays involving three or more groups a one-way ANOVA was used to analyze groups with Tukey post-test used to compare means of each group. All comparisons were considered significant at *P* < 0.05. GraphPad Prism was also used to generate standard curves with regression analyses from which values were calculated for sample measurements.

## SUPPLEMENTARY MATERIALS FIGURES AND VIDEO





## Abbreviations

Calcium: Ca^2+^
ER: endoplasmic reticulum
Grm1: glutamate receptor 1
IP3: inositol 1,4,5-trisphosphate
IP3R: inositol 1,4,5-trisphosphate receptor
SELENOK: Selenoprotein K
SOCE: store operated calcium entry
STIM: stromal interaction molecules
Tg: transgenic
Trp2: tyrosinase-related protein 2

## References

[1] Hogan PG, Rao A (2015). Store-operated calcium entry: Mechanisms and modulation. Biochem Biophys Res Commun.

[2] Zhou Y, Srinivasan P, Razavi S, Seymour S, Meraner P, Gudlur A, Stathopulos PB, Ikura M, Rao A, Hogan PG (2013). Initial activation of STIM1, the regulator of store-operated calcium entry. Nat Struct Mol Biol.

[3] Prakriya M, Feske S, Gwack Y, Srikanth S, Rao A, Hogan PG (2006). Orai1 is an essential pore subunit of the CRAC channel. Nature.

[4] Stathopulos PB, Ikura M (2016). Store operated calcium entry: From concept to structural mechanisms. Cell Calcium.

[5] Berridge MJ, Lipp P, Bootman MD (2000). The versatility and universality of calcium signalling. Nat Rev Mol Cell Biol.

[6] Villalobos C, Sobradillo D, Hernandez-Morales M, Nunez L (2016). Remodeling of Calcium Entry Pathways in Cancer. Adv Exp Med Biol.

[7] Cui C, Merritt R, Fu L, Pan Z (2017). Targeting calcium signaling in cancer therapy. Acta Pharm Sin B.

[8] Stanisz H, Vultur A, Herlyn M, Roesch A, Bogeski I (2016). The role of Orai-STIM calcium channels in melanocytes and melanoma. J Physiol.

[9] Verma S, Hoffmann FW, Kumar M, Huang Z, Roe K, Nguyen-Wu E, Hashimoto AS, Hoffmann PR (2011). Selenoprotein K knockout mice exhibit deficient calcium flux in immune cells and impaired immune responses. J Immunol.

[10] Meplan C, Johnson IT, Polley AC, Cockell S, Bradburn DM, Commane DM, Arasaradnam RP, Mulholland F, Zupanic A, Mathers JC, Hesketh J (2016). Transcriptomics and proteomics show that selenium affects inflammation, cytoskeleton, and cancer pathways in human rectal biopsies. FASEB J.

[11] Huang Z, Hoffmann FW, Norton RL, Hashimoto AC, Hoffmann PR (2011). Selenoprotein K is a novel target of m-calpain, and cleavage is regulated by Toll-like receptor-induced calpastatin in macrophages. J Biol Chem.

[12] Fredericks GJ, Hoffmann FW, Rose AH, Osterheld HJ, Hess FM, Mercier F, Hoffmann PR (2014). Stable expression and function of the inositol 1,4,5-triphosphate receptor requires palmitoylation by a DHHC6/selenoprotein K complex. Proc Natl Acad Sci U S A.

[13] Fredericks GJ, Hoffmann PR (2015). Selenoprotein K and protein palmitoylation. Antioxid Redox Signal.

[14] Hooper R, Zaidi MR, Soboloff J (2016). The heterogeneity of store-operated calcium entry in melanoma. Sci China Life Sci.

[15] Perotti V, Baldassari P, Bersani I, Molla A, Vegetti C, Tassi E, Dal Col J, Dolcetti R, Anichini A, Mortarini R (2012). NFATc2 is a potential therapeutic target in human melanoma. J Invest Dermatol.

[16] Schiffner S, Chen S, Becker JC, Bosserhoff AK (2012). Highly pigmented Tg(Grm1) mouse melanoma develops non-pigmented melanoma cells in distant metastases. Exp Dermatol.

[17] Pollock PM, Cohen-Solal K, Sood R, Namkoong J, Martino JJ, Koganti A, Zhu H, Robbins C, Makalowska I, Shin SS, Marin Y, Roberts KG, Yudt LM (2003). Melanoma mouse model implicates metabotropic glutamate signaling in melanocytic neoplasia. Nat Genet.

[18] Dou J, Pan M, Wen P, Li Y, Tang Q, Chu L, Zhao F, Jiang C, Hu W, Hu K, Gu N (2007). Isolation and identification of cancer stem-like cells from murine melanoma cell lines. Cell Mol Immunol.

[19] Cherciu I, Barbalan A, Pirici D, Margaritescu C, Saftoiu A (2014). Stem cells, colorectal cancer and cancer stem cell markers correlations. Curr Health Sci J.

[20] Wang C, Li R, Huang Y, Wang M, Yang F, Huang D, Wu C, Li Y, Tang Y, Zhang R, Cheng J (2017). Selenoprotein K modulate intracellular free Ca2+ by regulating expression of calcium homoeostasis endoplasmic reticulum protein. Biochem Biophys Res Commun.

[21] Hoffmann PR, Hatfield DL, Gladyshev VN, Berry MJ (2012). An emerging picture of the biological roles of selenoprotein K. Selenium: Its Molecular Biology and Role in Human Health.

[22] Umemura M, Baljinnyam E, Feske S, De Lorenzo MS, Xie LH, Feng X, Oda K, Makino A, Fujita T, Yokoyama U, Iwatsubo M, Chen S, Goydos JS (2014). Store-operated Ca2+ entry (SOCE) regulates melanoma proliferation and cell migration. PLoS One.

[23] Yang S, Zhang JJ, Huang XY (2009). Orai1 and STIM1 are critical for breast tumor cell migration and metastasis. Cancer Cell.

[24] Motiani RK, Hyzinski-Garcia MC, Zhang X, Henkel MM, Abdullaev IF, Kuo YH, Matrougui K, Mongin AA, Trebak M (2013). STIM1 and Orai1 mediate CRAC channel activity and are essential for human glioblastoma invasion. Pflugers Arch.

[25] Huang W, Ren C, Huang G, Liu J, Liu W, Wang L, Zhu B, Feng X, Shi J, Li J, Xia X, Jia W, Chen J (2017). Inhibition of store-operated Ca2+ entry counteracts the apoptosis of nasopharyngeal carcinoma cells induced by sodium butyrate. Oncol Lett.

[26] Hou MF, Kuo HC, Li JH, Wang YS, Chang CC, Chen KC, Chen WC, Chiu CC, Yang S, Chang WC (2011). Orai1/CRACM1 overexpression suppresses cell proliferation via attenuation of the store-operated calcium influx-mediated signalling pathway in A549 lung cancer cells. Biochim Biophys Acta.

[27] Hartman KG, Vitolo MI, Pierce AD, Fox JM, Shapiro P, Martin SS, Wilder PT, Weber DJ (2014). Complex formation between S100B protein and the p90 ribosomal S6 kinase (RSK) in malignant melanoma is calcium-dependent and inhibits extracellular signal-regulated kinase (ERK)-mediated phosphorylation of RSK. J Biol Chem.

[28] Dubois C, Vanden Abeele F, Lehen’kyi V, Gkika D, Guarmit B, Lepage G, Slomianny C, Borowiec AS, Bidaux G, Benahmed M, Shuba Y, Prevarskaya N (2014). Remodeling of channel-forming ORAI proteins determines an oncogenic switch in prostate cancer. Cancer Cell.

[29] Monteith GR (2014). Prostate cancer cells alter the nature of their calcium influx to promote growth and acquire apoptotic resistance. Cancer Cell.

[30] Pan Z, Ma J (2015). Open Sesame: treasure in store-operated calcium entry pathway for cancer therapy. Sci China Life Sci.

[31] Feldman B, Fedida-Metula S, Nita J, Sekler I, Fishman D (2010). Coupling of mitochondria to store-operated Ca(2+)-signaling sustains constitutive activation of protein kinase B/Akt and augments survival of malignant melanoma cells. Cell Calcium.

[32] Fiorio Pla A, Kondratska K, Prevarskaya N (2016). STIM and ORAI proteins: crucial roles in hallmarks of cancer. Am J Physiol Cell Physiol.

[33] Vervloessem T, Yule DI, Bultynck G, Parys JB (2015). The type 2 inositol 1,4,5-trisphosphate receptor, emerging functions for an intriguing Ca(2)(+)-release channel. Biochim Biophys Acta.

[34] Monteith GR, Prevarskaya N, Roberts-Thomson SJ (2017). The calcium-cancer signalling nexus. Nat Rev Cancer.

[35] Grosse J, Braun A, Varga-Szabo D, Beyersdorf N, Schneider B, Zeitlmann L, Hanke P, Schropp P, Muhlstedt S, Zorn C, Huber M, Schmittwolf C, Jagla W (2007). An EF hand mutation in Stim1 causes premature platelet activation and bleeding in mice. J Clin Invest.

[36] Oh-Hora M, Yamashita M, Hogan PG, Sharma S, Lamperti E, Chung W, Prakriya M, Feske S, Rao A (2008). Dual functions for the endoplasmic reticulum calcium sensors STIM1 and STIM2 in T cell activation and tolerance. Nat Immunol.

[37] Varga-Szabo D, Braun A, Kleinschnitz C, Bender M, Pleines I, Pham M, Renne T, Stoll G, Nieswandt B (2008). The calcium sensor STIM1 is an essential mediator of arterial thrombosis and ischemic brain infarction. J Exp Med.

[38] Hogan PG, Lewis RS, Rao A (2010). Molecular basis of calcium signaling in lymphocytes: STIM and ORAI. Annu Rev Immunol.

[39] Rose AH, Bertino P, Hoffmann FW, Gaudino G, Carbone M, Hoffmann PR (2014). Increasing dietary selenium elevates reducing capacity and ERK activation associated with accelerated progression of select mesothelioma tumors. Am J Pathol.

[40] Sulzmaier FJ, Young-Robbins S, Jiang P, Geerts D, Prechtl AM, Matter ML, Kesari S, Ramos JW (2016). RSK2 activity mediates glioblastoma invasiveness and is a potential target for new therapeutics. Oncotarget.

[41] Keese CR, Wegener J, Walker SR, Giaever I (2004). Electrical wound-healing assay for cells *in vitro*. Proc Natl Acad Sci U S A.

[42] Gawecka JE, Griffiths GS, Ek-Rylander B, Ramos JW, Matter ML (2010). R-Ras regulates migration through an interaction with filamin A in melanoma cells. PLoS One.

[43] Meiler S, Baumer Y, Huang Z, Hoffmann FW, Fredericks GJ, Rose AH, Norton RL, Hoffmann PR, Boisvert WA (2013). Selenoprotein K is required for palmitoylation of CD36 in macrophages: implications in foam cell formation and atherogenesis. J Leukoc Biol.

