# Correction: Selenoprotein K deficiency inhibits melanoma by reducing calcium flux required for tumor growth and metastasis

**DOI:** 10.18632/oncotarget.25857

**Published:** 2018-07-20

**Authors:** Michael P. Marciel, Vedbar S. Khadka, Youping Deng, Pascal Kilicaslan, Andrew Pham, Pietro Bertino, Katie Lee, Suzie Chen, Natalija Glibetic, FuKun W. Hoffmann, Michelle L. Matter, Peter R. Hoffmann

**Affiliations:** ^1^ Department of Cell and Molecular Biology, John A. Burns School of Medicine, University of Hawaii, Honolulu, Hawaii, U.S.A.; ^2^ Bioinformatics Core in the Department of Complementary and Integrative Medicine, John A. Burns School of Medicine, University of Hawaii, Honolulu, Hawaii, U.S.A.; ^3^ Biotechnology Department, University of Applied Sciences Mannheim, Mannheim, Germany; ^4^ Ernest Mario School of Pharmacy, Rutgers University, Piscataway, New Jersey, U.S.A.; ^5^ The University of Hawaii Cancer Center, Honolulu, Hawaii, U.S.A.

**This article has been corrected:** The corrected author name is given below:

**Youping Deng**

Original article: Oncotarget. 2018; 9:13407-13422. https://doi.org/10.18632/oncotarget.24388

