# Supplementary material for: Selenoprotein K deficiency inhibits melanoma by reducing calcium flux required for tumor growth and metastasis
Source: Oncotarget. 2018 Feb 3;9(17):13407–22. doi: 10.18632/oncotarget.24388 (PMC5862587; doi:10.18632/oncotarget.24388)
Supplement: Supplementary file 1 [file oncotarget-09-13407-s001.pdf]

Selenoprotein K deficiency inhibits melanoma by reducing calcium flux required for tumor growth and metastasis

SUPPLEMENTARY MATERIALS

Selenoprotein K immunohistology results

Patients with localized malignant melanoma	Patient 1	Patient 2	Patient 3	Patient 4	Patient 5
Normal tissue	1	1	1	0	0
Tumor tissue	1	1	0	0	0

Patients with distant or regional metastatic melanoma	Patient 1	Patient 2	Patient 3	Patient 4	Patient 5
Normal tissue	0	1	0	1	0
Tumor tissue	0	1	0	1	0

Glutathione peroxidase 1 immunohistology results

Patients with localized malignant melanoma	Patient 1	Patient 2	Patient 3	Patient 4	Patient 5
Normal tissue	2	2	2	1	2
Tumor tissue	2	2	2	2	2

Patients with distant or regional metastatic melanoma	Patient 1	Patient 2	Patient 3	Patient 4	Patient 5
Normal tissue	2	2	1	1	2
Tumor tissue	2	2	1	1	2

Glutathione peroxidase 4 immunohistology results

Patients with localized malignant melanoma	Patient 1	Patient 2	Patient 3	Patient 4	Patient 5
Normal tissue	1	2	1	1	1
Tumor tissue	2	2	1	1	1

Patients with distant or regional metastatic melanoma	Patient 1	Patient 2	Patient 3	Patient 4	Patient 5
Normal tissue	1	1	1	1	1
Tumor tissue	2	2	1	2	1

**Scale**	
0	Negative
1	Positive - Weak in at least 25% of the cells
2	Positive - Medium in at least 25% of the cells
3	Positive - Strong in at least 25% of the cells

Supplementary Figure 1: Results from immunohistochemical (IHC) evaluation of early and late stage melanoma tissues obtained from the University of Hawaii Cancer Center (UHCC). IHC was performed and scored by the UHCC Pathology core for SELENOK along with two other selenoproteins, glutathione peroxidase-1 and -4. Results suggest no changes in SELENOK levels in tumor tissues compared to normal tissue.

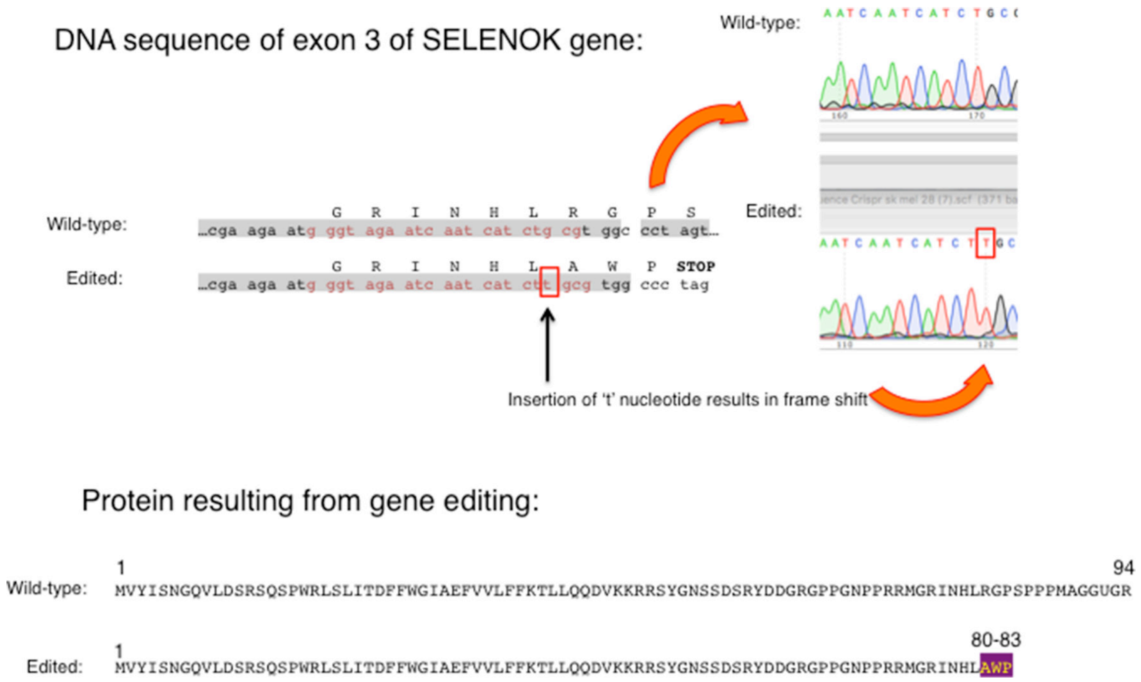

Supplementary Figure 2: Sanger sequencing results for edited clones compared to w.t. SK-MEL-28 melanoma cells. CRISPR/Cas9 editing led to introduction of a thymidine nucleotide causing a frame shift in DNA. This resulted in altered codons corresponding to amino acids 80–83 followed by a stop codon leading to a truncated SELENOK protein lacking the functional domain.

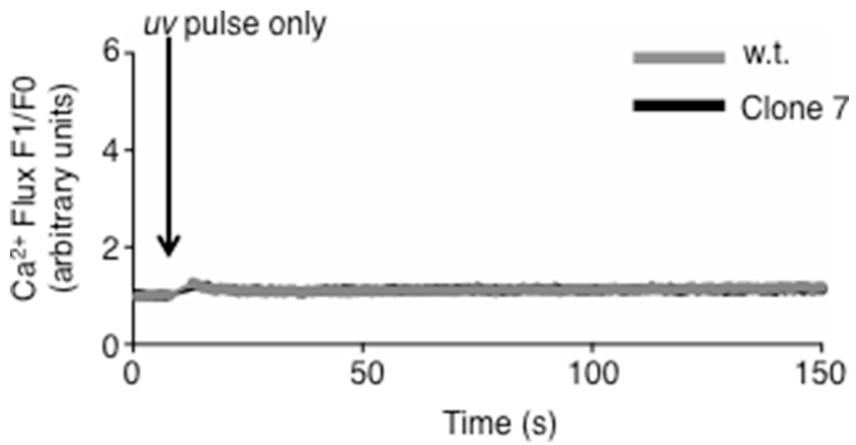

Supplementary Figure 3: SELENOK-deficient cells (Clone 7) compared to wild-type SK-MEL-28 cells were loaded with FuraRed along with no caged IP3 to determine effects of the 2 s *uv* pulse on Ca^{2+} flux. No caged IP3 showed minimal Ca^{2+} flux, which indicates uv alone does not induce a response.

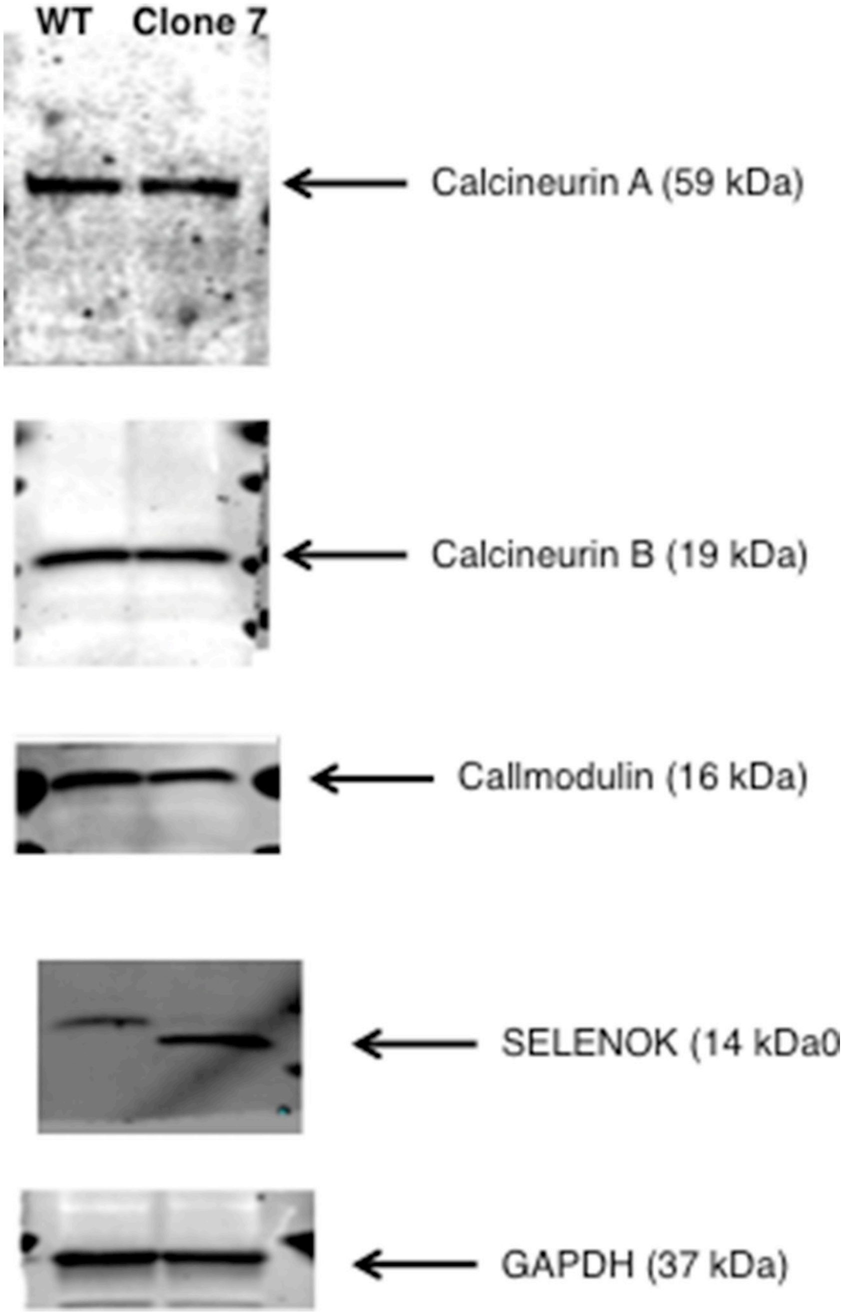

Supplementary Figure 4: Western blotting was performed to determine levels of calcineurin subunits A and B, along with calmodulin in SELENOK-deficient cells (Clone 7) compared to wild-type SK-MEL-28 cells. Results showed no differences. SELENOK was detected in Clone 7 cells as a smaller truncated form as expected and GAPDH was used as a loading control.

Gene Symbol	Fold Change	FDR-adjusted p-value	Average Expression for KO (N=3)	Average Expression for Wild-Type (N=3)
ADGRL2	2.2807	0.0237	8.2676	7.0781
DCAF6	2.0584	0.0205	9.8599	8.8184
ATP1B1	2.1094	0.0139	9.9843	8.9074
DNM3	2.6882	0.0247	5.9884	4.5618
PRELP	2.1109	0.0247	8.1306	7.0528
DYRK3	2.7438	0.0131	9.1398	7.6837
KCNK2	2.1077	0.0217	7.2237	6.148
EPHX1	2.0405	0.0101	10.2165	9.1876
LGALS8	2.6707	0.0072	9.6015	8.1843
NEGR1	3.9401	0.0179	7.9467	5.9685
TXNIP	2.3673	0.0101	11.0101	9.7669
CTSK	2.4215	0.0155	10.8837	9.6078
SPRR2D	3.3653	0.0072	10.0731	8.3224
SPRR2B	2.1712	0.034	6.7953	5.6768
S100A6	3.8477	0.0165	8.1262	6.1823
SLAMF9	3.1588	0.0252	9.0723	7.4129
GPR161	2.0635	0.0363	9.0948	8.0497
OPN3	2.0396	0.0232	7.7807	6.7524
CNIH3	5.6007	0.0077	9.7801	7.2944
F11R	2.3484	0.0108	10.0395	8.8078
INSIG2	2.3873	0.0108	9.3963	8.1409
CDK15	3.009	0.0101	6.5941	5.0049
MAP2	2.7741	0.0097	9.473	8.001
MGAT4A	2.8635	0.0168	6.2607	4.7429
ZRANB3	2.3576	0.0491	6.7114	5.4741
GPR155	2.7864	0.0197	9.2266	7.7482
TFPI	2.0981	0.0234	6.9306	5.8615
HIBCH	2.2895	0.0449	9.0288	7.8338
KLF7	2.1725	0.0128	10.2687	9.1493
DNER	4.1277	0.0072	7.9208	5.8755
TTN	2.0928	0.0308	7.3677	6.3023
TMEM45A	2.4449	0.0116	9.3116	8.0218
CD96	2.3951	0.0182	5.2435	3.9834
EEFSEC	2.0821	0.0049	9.3114	8.2534
NEK11	2.255	0.03	6.0446	4.8714
PPP2R3A	2.6855	0.0193	9.3027	7.8775
SUCNR1	3.5176	0.0101	5.0813	3.2667
MME	2.1991	0.049	9.9928	8.8559
KCNAB1	2.1249	0.0174	8.3712	7.2837
GPR160	3.1847	0.0185	7.7971	6.1259
NLGN1	2.1464	0.0131	9.6067	8.5048
LMLN	2.4709	0.017	9.5689	8.2639
OSBPL10	4.81	0.012	9.9677	7.7017
CADPS	6.2635	0.0072	9.2556	6.6086
EIF4E3	2.8319	0.0152	7.5262	6.0244
CCDC191	2.4902	0.0256	6.592	5.2758
MIR568///ZBTB2	2.39	0.0247	10.1811	8.9241
LSAMP	2.4976	0.0221	9.5593	8.2388
RABL3	2.1159	0.0152	8.4078	7.3265
WDR5B	2.2071	0.0108	7.2282	6.086
ITGB5	2.041	0.0134	10.3882	9.3589
TMCC1	2.0858	0.0239	7.4893	6.4287
STAG1	2.0267	0.0131	9.701	8.6819
SIAH2	2.4326	0.0158	10.2955	9.013
SLC33A1	2.1224	0.0108	9.1991	8.1134
SHOX2	2.2856	0.0293	9.0587	7.8661
PEX5L	3.3716	0.0236	5.6481	3.8947
KLHL6	2.5185	0.0077	7.2063	5.8737
APOD	2.6987	0.0108	12.496	11.0637
MFI2	2.2974	0.0108	11.4673	10.2673
ROBO2	5.4042	0.0117	7.7618	5.3278
C3orf58	2.1744	0.0185	9.3254	8.2048
SATB1	3.8315	0.0205	7.4022	5.4643
ACAD11	2.0288	0.0401	7.5886	6.568
PIK3CB	2.1097	0.0476	8.8519	7.7748
TM4SF19-TCTEX1I	2.8455	0.0286	7.7701	6.2614
TM4SF19	2.7132	0.0174	7.13	5.69
FAM114A1	2.2862	0.0387	7.6269	6.4339
UGT2B7	3.4312	0.0393	8.1026	6.3239

Gene Symbol	Fold Change	FDR-adjusted p-value	Average Expression for KO (N=3)	Average Expression for Wild-Type (N=3)
SPP1	5.7314	0.0072	11.9714	9.4525
PPARGC1A	2.1654	0.0297	8.7152	7.6006
FGFBP2	3.1926	0.0373	8.0571	6.3824
TLR6	3.8531	0.025	6.7374	4.7914
ITGA2	2.0787	0.0157	8.1915	7.1358
OCLN	2.1705	0.0261	7.0974	5.9794
ERAP2	4.1572	0.0155	8.6709	6.6153
CYFIP2	2.248	0.0244	8.008	6.8393
ARRDC3	2.8832	0.0325	8.1334	6.6058
EPB41L4A	4.3126	0.0323	5.2865	3.1779
NME5	3.4101	0.0179	5.5883	3.8184
ATP6AP1L	2.1013	0.0301	9.1171	8.0458
AIG1	2.1416	0.0108	8.619	7.5204
NRN1	2.174	0.0359	10.3592	9.2388
GPR63	2.004	0.0192	5.6917	4.6888
FSCN1	2.5297	0.0182	9.2712	7.9322
ABCB5	6.7511	0.0172	11.4473	8.6921
BBS9	2.0731	0.027	9.0365	7.9847
ADAM22	2.422	0.0239	8.0015	6.7253
STEAP2	6.6195	0.0217	6.8075	4.0807
GNG11	2.3345	0.0072	8.5912	7.3681
SLC26A4	3.3053	0.0303	7.2559	5.5312
MET	3.6531	0.0143	10.0205	8.1514
CPVL	2.5626	0.0072	9.5088	8.1512
PCLO	2.134	0.0424	9.0218	7.9283
SAMD9L	2.6725	0.0217	8.8234	7.4052
DGKI	2.3657	0.0174	6.9665	5.7242
KIAA1147	2.0439	0.0352	8.4954	7.4641
ZDHHC2	4.5963	0.0257	7.0611	4.8607
MATN2	3.2426	0.0243	7.5443	5.8472
NOV	2.8049	0.0191	8.6448	7.1569
PSCA	3.5805	0.0128	11.6077	9.7675
CSGALNACT1	2.0664	0.0414	7.1794	6.1322
CLU///MIR6843	2.1107	0.0155	6.1345	5.0568
ENPP2	3.251	0.0168	8.2555	6.5546
NDRG1	2.6091	0.0077	10.0125	8.6289
XKR9	2.1392	0.0239	6.1571	5.06
RORB	2.5193	0.0113	9.1196	7.7866
NMRK1	2.22	0.0155	7.7364	6.5858
GKAP1	2.3345	0.0108	4.8653	3.6421
BAAT	10.3718	0.0131	8.0309	4.6563
CS	2.4045	0.0252	7.4223	6.1566
PGM5	2.4344	0.0232	7.1669	5.8834
HTR2C	2.2822	0.0492	6.4228	5.2324
STK26	2.0534	0.0239	8.5742	7.5362
MAGEC1	9.4116	0.0108	9.1958	5.9613
DMD	2.2421	0.0174	5.5526	4.3878
PLXDC2	10.3874	0.0106	8.2365	4.8598
DYDC2	2.6771	0.0152	7.6098	6.1891
PNLIPRP3	6.3812	0.0152	9.1921	6.5183
FAM188A	2.3401	0.0374	9.1417	7.9151
SORBS1	2.14	0.0384	8.0507	6.9531
ABLIM1	2.0899	0.0263	6.4611	5.3977
ENTPD1	3.3319	0.0182	9.9632	8.2268
LIPA	2.5618	0.0207	11.0887	9.7316
CRYAB	12.8729	0.0072	8.812	5.1257
TNS2	2.367	0.0493	8.1183	6.8813
SLC16A7	2.7268	0.018	7.6046	6.1574
LYZ	4.8877	0.0152	7.341	5.0518
GUPR1	2.3152	0.0313	8.4478	7.2367
FBXW8	2.0531	0.0195	9.6973	8.6595
A2M	3.0851	0.0254	9.4668	7.8415
PZP	2.0375	0.025	7.6095	6.5827
GRIP1	2.0571	0.0199	6.9554	5.9148
PTPRR	5.0781	0.019	7.7533	5.409
ALDH1L2	3.9757	0.0108	8.684	6.6928
RDH5	2.3025	0.0108	8.5526	7.3494
HSPB8	2.3061	0.0117	10.0103	8.8049
GPC6	2.0197	0.0131	9.9082	8.8941

Gene Symbol	Fold Change	FDR-adjusted p-value	Average Expression for KO (N=3)	Average Expression for Wild-Type (N=3)
NALCN	6.2481	0.0258	7.7448	5.1014
NRXN3	2.4762	0.03	7.4283	6.1201
AHNAK2	2.5997	0.0213	8.0619	6.6835
GABRA5	6.0134	0.018	8.5406	5.9524
ATP10A	2.399	0.016	8.8294	7.5669
CCPG1///MIR62	2.0743	0.0346	9.1202	8.0675
ZDHHC1	3.577	0.043	6.6363	4.7975
GPRC5B	2.505	0.0298	7.9126	6.5877
GPRC5B	2.2527	0.0214	10.2788	9.1072
SEPT4	6.2539	0.0072	8.8872	6.2425
CD68	2.4689	0.0174	12.5362	11.2313
RAB27B	2.7412	0.0173	8.4089	6.9541
CDH7	2.595	0.0131	7.8463	6.4705
CHST9	6.4049	0.0136	6.4823	3.8032
CDH19	3.4663	0.0155	7.1682	5.3748
GDF15	4.9795	0.0049	11.728	9.412
FXYD3///MIR68	6.8627	0.0042	12.6998	9.921
CD22///MIR519	2.2476	0.0438	8.7198	7.5514
RCN3	2.2405	0.0049	10.8491	9.6853
SEMA6B	2.4734	0.0216	8.3394	7.0329
OLFM2	2.0216	0.0213	8.3136	7.2981
BST2	6.458	0.0131	9.4402	6.7491
PLAUR	2.1155	0.0132	10.6004	9.5193
ZNF776	2.0375	0.0207	7.8075	6.7808
C79160///RP5-90	3.1156	0.035	7.901	6.2615
IFT27	2.0899	0.0403	7.9816	6.9182
ZNF546	2.1811	0.0279	4.2165	3.0915
ZNF546	2.3453	0.0303	7.1851	5.9553
ZYG11A	-2.6941	0.0152	4.2347	5.6646
EDARADD	-2.4679	0.037	4.8462	6.1495
EPHA2	-2.4275	0.0246	5.9842	7.2637
TMCO4	-2.8551	0.0108	6.617	8.1306
RAP1GAP	-3.8761	0.0108	5.2549	7.2095
LAPTM5	-2.3652	0.0465	6.2283	7.4702
SYNC	-2.1773	0.0398	5.3711	6.4936
HENMT1	-3.776	0.0279	5.7262	7.6431
CTSS	-2.0543	0.0143	6.0388	7.0774
RXRG	-2.4538	0.0174	8.3152	9.6102
///ZNF670-ZNF69	-3.1027	0.0108	6.1316	7.7651
KLHL29	-4.5666	0.0166	4.8293	7.0205
ARHGAP25	-2.287	0.0367	7.1085	8.302
POTEE	-7.823	0.0132	3.867	6.8347
B3GALT1	-2.3967	0.037	4.3889	5.6499
CERS6	-2.3532	0.0414	7.7863	9.0209
ICOS	-8.8068	0.0058	2.9203	6.0589
ADAM23	-3.3993	0.0138	8.1925	9.9577
FAR2P1	-4.155	0.0285	4.7174	6.7723
POTEF	-11.6334	0.0131	3.8913	7.4315
POTEI	-6.4517	0.0217	3.0746	5.7642
GRB14	-2.9486	0.0453	4.3349	5.8949
RFTN1	-2.2282	0.0114	6.5773	7.7332
SUSD5	-2.4041	0.0336	8.0036	9.269
MGLL	-5.7699	0.0072	6.0354	8.5639
B3GALNT1	-5.216	0.018	3.9567	6.3396
EPHB1	-2.9112	0.0143	6.4913	8.0329
D4S234E///NSG	-3.8492	0.0131	7.1153	9.0598
GALNT7	-9.658	0.0108	4.7724	8.0441
CDKL2	-2.2784	0.0393	2.9971	4.1852
PRKG2	-5.7786	0.0079	6.6367	9.1674
ABCG2	-2.0484	0.0386	4.0828	5.1173
AADAT	-2.3001	0.0393	7.1677	8.3694
PROM1	-12.0814	0.0106	5.7861	9.3808
GHR	-3.3539	0.0185	6.349	8.0948
EMB	-4.1267	0.0108	6.496	8.541
SLIT3	-6.2522	0.0152	5.6691	8.3135
LCP2	-3.3438	0.0197	7.5649	9.3064
HLA-DRA	-2.2397	0.0174	8.4521	9.6154
MRAP2	-4.2025	0.0097	5.5886	7.6598
HLA-DQB1	-2.9943	0.0435	6.9657	8.5479

Gene Symbol	Fold Change	FDR-adjusted p-value	Average Expression for KO (N=3)	Average Expression for Wild-Type (N=3)
MB21D1	-2.9445	0.0383	5.1799	6.7379
SRSF12	-3.0703	0.0108	7.6216	9.24
ZNF716	-5.662	0.0237	5.4255	7.9269
AMPH	-2.566	0.0313	6.3068	7.6663
FABP5	-2.317	0.0112	7.9223	9.1346
RIMS2	-2.155	0.0307	4.7543	5.862
EGR3	-2.9975	0.0174	6.9083	8.4921
TRPA1	-2.2263	0.0461	6.0457	7.2004
EXT1///spawla	-2.3109	0.0336	6.2572	7.4656
HACD4	-3.1423	0.0174	7.2737	8.9255
SHB	-3.0477	0.0174	7.5115	9.1192
MAOA	-3.4543	0.0247	5.8037	7.5921
TRO	-4.7652	0.0198	4.2424	6.4949
LONRF3	-13.3226	0.0131	4.8886	8.6244
WNK3	-2.5889	0.0133	5.1755	6.5478
TSPAN7	-2.2004	0.0242	8.0247	9.1625
MGMT	-2.4919	0.0205	5.1718	6.4891
SFMBT2	-3.5509	0.0215	4.5146	6.3428
SORCS1	-2.1483	0.0131	7.4842	8.5874
ZNF215	-2.755	0.012	6.2579	7.72
HTATIP2	-2.0274	0.0232	8.344	9.3636
DEPDC7	-2.0255	0.0236	6.4523	7.4706
NTM	-10.4911	0.0312	4.851	8.2421
IFITM3	-4.0626	0.007	9.2065	11.2289
PHLDA2	-2.2258	0.0166	7.787	8.9414
MPPED2	-2.6801	0.0312	5.9869	7.4092
FABP5	-2.1628	0.011	6.9339	8.0468
SF1	-2.1936	0.0431	6.3596	7.4927
SYTL2	-2.4765	0.0072	9.3041	10.6124
MMP1	-13.467	0.0108	7.9878	11.7392
IFITM2	-2.9441	0.0165	7.3113	8.8692
IFITM1	-2.2926	0.0211	6.6046	7.8017
SPX	-2.4296	0.0232	3.7321	5.0127
CNTN1	-2.6048	0.0323	7.9974	9.3785
CRACR2A	-2.8538	0.0138	6.6565	8.1694
SLC38A1	-10.0496	0.0108	3.2034	6.5325
TRPV4	-3.3185	0.0101	8.9501	10.6806
LINC00282	-2.8412	0.0333	8.624	10.1305
TBC1D4	-2.356	0.0303	8.3984	9.6348
DNAJC15	-6.8111	0.007	7.3472	10.1151
CRIP2	-2.0263	0.0143	7.4465	8.4653
FLVCR2	-2.1524	0.0339	5.1418	6.2477
DNAJA4	-2.767	0.0257	6.2322	7.7006
ACAN	-2.7991	0.0465	6.448	7.9329
LRRK1	-2.1226	0.0407	8.1143	9.2002
CYP1A1	-2.6169	0.0153	5.8029	7.1908
XYLT1	-3.5821	0.0182	6.7721	8.6129
YPEL3	-2.3449	0.0437	6.7047	7.9342
ACSM3	-2.0116	0.0181	5.8211	6.8295
TMEM98	-2.0987	0.0072	9.4901	10.5596
MEIOC	-2.8785	0.0445	4.1346	5.6599
SPATA22	-6.119	0.0174	5.1691	7.7824
RAB31	-4.5799	0.0149	5.2216	7.4169
SETBP1	-2.3802	0.0211	6.6988	7.9498
ZNF382	-4.6534	0.0249	4.9538	7.172
ZNF114	-10.0347	0.0077	7.2847	10.6116
PRDX2	-15.045	0.0152	5.658	9.5692
ZNF43	-2.1063	0.0354	8.1934	9.2681
ZNF724P	-2.138	0.0239	7.1217	8.2179
ZNF611	-2.7692	0.0345	7.728	9.1975
ZNF888	-2.4762	0.0293	4.8818	6.1899
ZNF730	-6.0707	0.0379	5.5623	8.1641
RPL39P34///ZNF8C	-2.5093	0.0131	7.0385	8.3658
ZNF582-AS1	-2.8685	0.0405	5.2119	6.7322
ZNF607	-2.2272	0.0135	4.3353	5.4905
ZNF28	-9.0467	0.0101	4.6105	7.7878
ZNF816	-2.4499	0.0477	5.7192	7.0119
MYL9	-4.1723	0.017	6.9966	9.0675
BMP7	-2.3585	0.023	7.9222	9.1601
MX1	-2.645	0.0279	6.5742	7.9775
PDXP///SH3BP1	-2.035	0.0101	9.0256	10.0507
BAIAP2L2	-2.663	0.0129	7.7508	9.1638

Supplementary Figure 5: Differentially expressed genes in Clone 7 with respect to w.t. SK-MEL-28 melanoma cells along with fold-change (red = up-regulated; green = down-regulated), Benjamine & Hochberg FDR-adjusted *p*-value and average gene expressions.

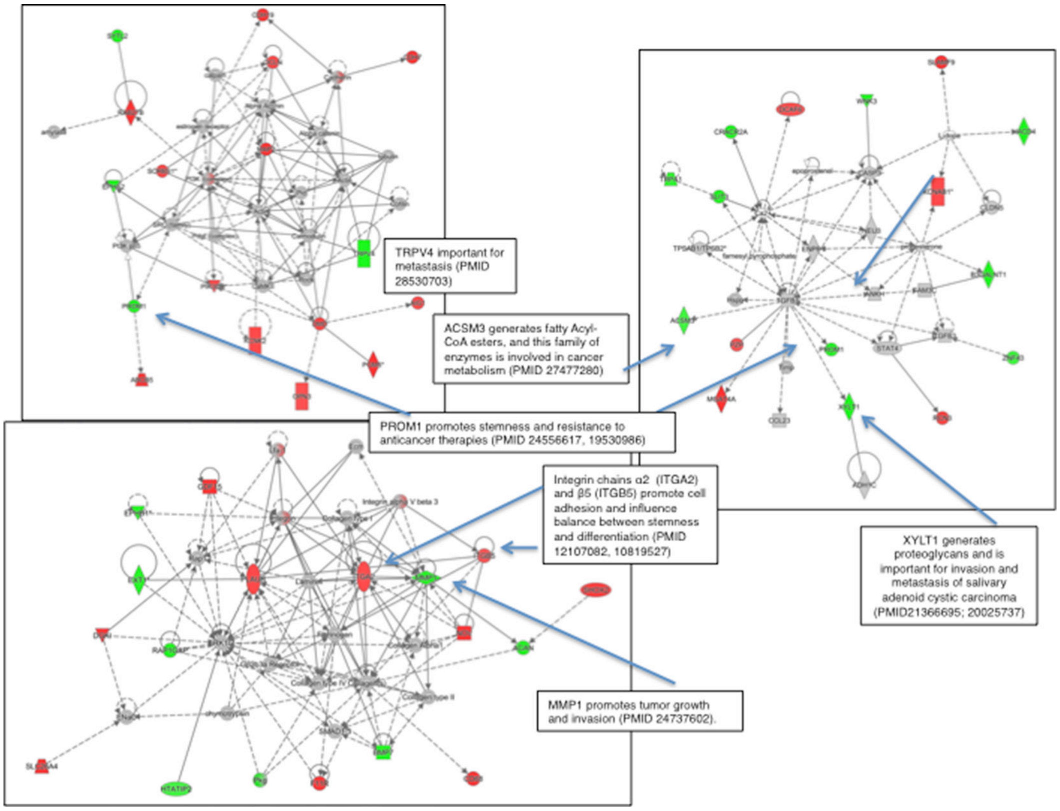

Supplementary Figure 6: Signaling pathway analyses conducted with QIAGEN's Ingenuity pathway analysis (Ingenuity Systems). Green indicates down-regulated transcripts and red indicates up-regulated transcripts in SELENOK-deficient cells compared to wild-type SK-MEL-28 cells.

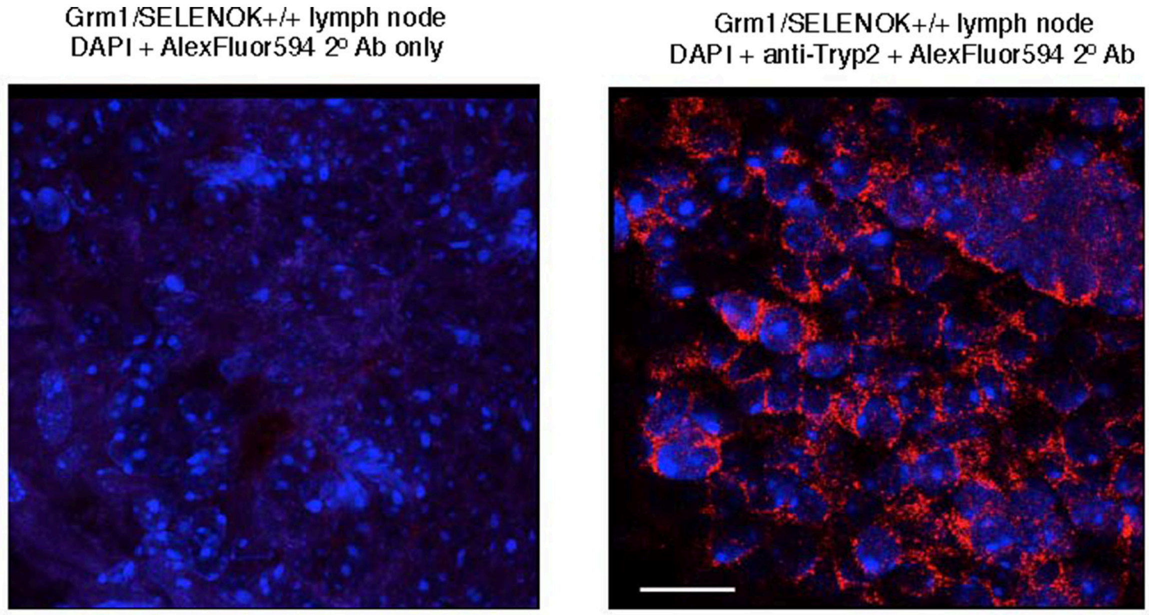

Supplementary Figure 7: Specific immunofluorescence staining using anti-Trp2, scalebar = 20 µm. Blue staining are DAPI-positive nuclei and red cytosolic staining is Trp2 antigen. Supplementary video 1 shows mice at 6 months of age that exhibit a lack of melanoma (Grm1-Tg/SELENOK$^{-/-}$) or high levels of melanoma (Grm1-Tg/SELENOK$^{-/+}$). As shown in the video, the mice with high tumor burden are not severely affected in terms of movement or healthy behavior. Several cohorts of both sexes were followed out to the age of 8 months and no increased tumor formation were found in the Grm1-Tg/SELENOK$^{-/-}$ mice.

Supplementary Video 1: Video showing tumors in 8 month-old mouse. See Supplementary_Video 1
